# Cellular Regulation of Amyloid Formation in Aging and Disease

**DOI:** 10.3389/fnins.2017.00064

**Published:** 2017-02-14

**Authors:** Esther Stroo, Mandy Koopman, Ellen A. A. Nollen, Alejandro Mata-Cabana

**Affiliations:** European Research Institute for the Biology of Aging, University of Groningen, University Medical Center GroningenGroningen, Netherlands

**Keywords:** neurodegeneration, protein aggregation, amyloid, protein quality control, SERF

## Abstract

As the population is aging, the incidence of age-related neurodegenerative diseases, such as Alzheimer and Parkinson disease, is growing. The pathology of neurodegenerative diseases is characterized by the presence of protein aggregates of disease specific proteins in the brain of patients. Under certain conditions these disease proteins can undergo structural rearrangements resulting in misfolded proteins that can lead to the formation of aggregates with a fibrillar amyloid-like structure. Cells have different mechanisms to deal with this protein aggregation, where the molecular chaperone machinery constitutes the first line of defense against misfolded proteins. Proteins that cannot be refolded are subjected to degradation and compartmentalization processes. Amyloid formation has traditionally been described as responsible for the proteotoxicity associated with different neurodegenerative disorders. Several mechanisms have been suggested to explain such toxicity, including the sequestration of key proteins and the overload of the protein quality control system. Here, we review different aspects of the involvement of amyloid-forming proteins in disease, mechanisms of toxicity, structural features, and biological functions of amyloids, as well as the cellular mechanisms that modulate and regulate protein aggregation, including the presence of enhancers and suppressors of aggregation, and how aging impacts the functioning of these mechanisms, with special attention to the molecular chaperones.

## Introduction

The process of aging is defined as a time-dependent functional decline eventually resulting in an increased vulnerability to death (reviewed in López-Otín et al., 2013). Gaining knowledge about the molecular events that occur in the cell during aging is important in order to understand the disease process of age-related diseases. Some neurodegenerative diseases, including Alzheimer (AD), Parkinson (PD), and Huntingtin disease (HD), share as hallmark the appearance of protein aggregates with fibrillary amyloid-like structures in the brain. These amyloid fibrils are composed of aggregation-prone proteins, such as mutant huntingtin (HTT) in Huntington disease, α-synuclein in Parkinson disease, and amyloid-beta (Aβ) in Alzheimer disease (Scherzinger et al., 1999; Chiti and Dobson, 2006; Goedert and Spillantini, 2006; See Table 1 for a list of aggregation-prone proteins involved in neurodegenerative diseases). The role of these aggregates in disease is not fully understood: the most prevalent hypothesis is that aggregation intermediates—single or complexes of aggregation-prone proteins—are toxic to cells and that the aggregation process represents a cellular protection mechanism against these toxic intermediates (Lansbury and Lashuel, 2006; Hartl and Hayer-Hartl, 2009).

**Table 1:** **Neurodegenerative diseases associated with protein aggregation**.

	**Identified disease genes**	**Protein that aggregates**	**Location of aggregates**	**Affected brain region**
Alzheimer disease (AD)	*APP* (Chartier-Harlin et al., 1991; Goate et al., 1991; Murrell et al., 1991)	Amyloid-beta, Tau	Extracellular	Cortex and Hippocampus
	*PS1* (Sherrington et al., 1995)		Intracellular	
	*PS2* (Levy-Lahad et al., 1995; Rogaev, 1995)			
Huntington disease (HD)	*HD* (Hess et al., 2016)	Huntingtin	Intracellular	Striatum
Parkinson disease (PD)	*SNCA* (Polymeropoulos et al., 1997)	Alpha synuclein	Intracellular	Substantia Nigra
	*Parkin* (Kitada et al., 1998)			
	*PINK1* (Valente et al., 2001)			
	*DJ1* (Bonifati et al., 2003)			
	*LRRK* (Zimprich et al., 2004) e.a.			
Dementia with Lewy bodies (DLB)	*SNCA* (Higuchi et al., 1998)	Alpha synuclein	Intracellular	Cortex and hippocampus
	*SNCB* (Ohtake et al., 2004)			
Frontotemporal dementia (FTA)	*MAPT* (Wilhelmsen et al., 1994)	Tau	Intracellular	Frontal and temporal cortex
Prion disease (PrD)	*PRNP* (Oesch et al., 1985)	Prion protein	Extracellular	Brain and spinal cord
Amyotrophic lateral sclerosis (ALS)	*SOD1* (Rosen et al., 1993)	SOD, FUS, TDP-43	Intracellular	Upper and lower Motor neurons
	*FUS* (Kwiatkowski et al., 2009)			
	*C9orf72* (DeJesus-Hernandez et al., 2011; Renton et al., 2011) e.a.			

The familial forms of many neurodegenerative diseases appear to involve toxic gain-of-function mutations in disease-specific proteins that increase their misfolding and aggregation properties. The resulting misbalance in protein homeostasis can speed up the process of amyloid formation, thereby often provoking an early-onset of several neurodegenerative disorders.

In this review, we address the involvement of aggregation-prone proteins in the development of different age-related disease. We describe how different cellular regulators impact on protein aggregation and how they are affected by aging, with special focus on the molecular chaperone machinery and other pathways involved in maintaining protein homeostasis. We also discuss different mechanisms that may underlie the toxicity of amyloid-forming proteins and we highlight some new findings in the amyloid field.

## Cellular regulators of protein aggregation

### Protein quality control

Cells have a protein quality control (PQC) system to maintain protein homeostasis. Preserving protein homeostasis involves the coordinated action of several pathways that regulate biogenesis, stabilization, correct folding, trafficking, and degradation of proteins, with the overall goal to prevent the accumulation of misfolded proteins and to maintain the integrity of the proteome.

### Chaperones

One of the cellular mechanisms that copes with misfolded proteins is the chaperone machinery. A molecular chaperone is defined as a protein that interacts with, stabilizes or assists another protein to gain its native and functionally active conformation without being present in the final structure (Ellis, 1987). Many members of the chaperone protein family are referred to as heat shock proteins (HSP), as they are upregulated during stress conditions such as heat shock (Ellis and Hartl, 1999; Kim et al., 2013). In addition to folding of misfolded proteins, molecular chaperones are also involved in a wide range of biological processes such as the folding of newly synthesized proteins, transport of proteins across membranes, macromolecular-complex assembly or protein degradation and activation of signal transduction routes (Kim et al., 2013; Kakkar et al., 2014). Under the denomination of “molecular chaperones” there are a variability of proteins that have been classified into five different families according to sequence homology, common functional domains or subcellular localization: the HSP100s, the HSP90s, the HSP70/HSP110, HSP60/CCTs, and the a-crystallin-containing domain generally called the “small HSPs” (Lindquist and Craig, 1988; Sharma and Priya, 2016). Typically, molecular chaperones recognize exposed hydrophobic domains in unfolded or misfolded proteins, preventing their self-association and aggregation (Hartl et al., 2011; Kim et al., 2013). The regulation of chaperones can be divided into three categories, (1) constitutively expressed, (2) induced upon stress, and (3) constitutively expressed and induced upon stress (Morimoto, 2008). Under normal conditions the HSP levels match the overall level of protein synthesis, but during stress when mature proteins are unfolded the chaperone machinery is challenged and the expression of specific HSPs increases (Kakkar et al., 2014).

Next to their function under normal cellular conditions, chaperones play an important part during neurodegeneration when there is an overload of the PQC system by unfolded proteins (Kim et al., 2013; Kakkar et al., 2014; Lindberg et al., 2015). Each neurodegenerative disease is associated with a different subset of HSPs that can positively influence the overload of unfolded proteins (Kakkar et al., 2014). One example is the molecular chaperone DNAJB6b that can suppress polyglutamine (polyQ) aggregation and toxicity in a cell model for polyQ diseases (Hageman et al., 2010; Gillis et al., 2013), and suppress the primary nucleation step by a direct protein-protein interaction with polyQ proteins (Månsson et al., 2014b) and Aβ42 (Månsson et al., 2014a). Overexpression of DNAJB6 in a mouse model for HD results in reduction of the disease symptoms and increase life span (Kakkar et al., 2016). In PD, the overexpression of HSP70 can prevent α-synclein-induced cell death in yeast, *Drosophila* and mouse models of this disease (Auluck and Bonini, 2002; Klucken et al., 2004; Flower et al., 2005; Sharma and Priya, 2016). HSP70 has been shown to bind prefibrillar species of α-synclein and to inhibit the fibril formation (Dedmon et al., 2005). There is also a role for molecular chaperones in AD, where the overexpression of heat shock factor 1 (HSF-1), main regulator of HSPs expression, in an AD mouse model diminished soluble Aβ levels (Pierce et al., 2013), and multiple HSPs alleviated Tau toxicity in cells (Kakkar et al., 2014).

Additionally to the inhibition of protein aggregation of misfolded proteins, a disaggregase activity has been described for some molecular chaperones that can solubilize aggregated proteins (Glover and Lindquist, 1998; Tyedmers et al., 2010; Winkler et al., 2012). In bacteria, yeast, fungi and plants the HSP100 disaggregases are highly conserved (Tyedmers et al., 2010; Torrente and Shorter, 2013). In yeast, HSP104 collaborates with the other HSPs, to effectively disaggregate and reactivate proteins trapped in disordered aggregates (Glover and Lindquist, 1998; Shorter, 2011; Torrente and Shorter, 2013; Lindberg et al., 2015). Metazoans entirely lack HSP100 disaggregases in the cell, however, it has recently shown that in mammalians the disaggregase function is performed by the HSPH (Hsp110) family in cooperation with the HSP70-40 machine (Rampelt et al., 2012; Gao et al., 2015; Nillegoda and Bukau, 2015). This machinery has been shown to fragmentize and depolarize large α-synclein fibrils within minutes into smaller fibrils, oligomers and monomeric α-synclein in an ATP-dependent fashion (Gao et al., 2015).

Chaperones are also involved in other pathways of PQC. As discussed below they can mediate the degradation of misfolded proteins or their sequestration in cellular compartments.

Together, this shows the important direct role chaperones play in the formation of amyloids and thereby making chaperones an interesting therapeutic target for neurodegenerative diseases.

### Protein degradation

Protein degradation is another key mechanism to deal with misfolded proteins. Three pathways have been described, i.e., the ubiquitin (Ub)-proteasome system (UPS), chaperone mediated autophagy (CMA), and macroautophagy (Ciechanover, 2006; Ciechanover and Kwon, 2015). Soluble misfolded proteins are degraded by the UPS, a system that is dependent on a cascade of three enzymes E1, E2, and E3 ligase that conjugate ubiquitin to the misfolded proteins. The ubiquitinated protein is transported by molecular chaperones to the proteolytic system, where the protein is unfolded and passed through the narrow chamber of the proteasome that cleaves it into short peptides (Ciechanover et al., 2000). The CMA degrades proteins that expose KFERQ-like regions, these regions are recognized by the chaperone heat-shock cognate 70 (Hsc70) and delivered to the lysosomes and degraded by lysosomal hydrolases into amino acids (Kiffin et al., 2004; Rothenberg et al., 2010). Protein aggregates or proteins that escape the first two degradation pathways are directed to macroautophagy, a degradation system where substrates are segregated into autophagosomes which in turn are fused with lysosomes for degradation into amino acids (Koga and Cuervo, 2011). The proteins involved in neurodegenerative disease can rapidly aggregate and can thereby escape degradation when they are still soluble, the aggregates, and intermediate forms are partly resistant to the known degradation pathways (reviewed in Ciechanover and Kwon, 2015).

### Unfolded protein response

In the endoplasmic reticulum (ER), the unfolded protein response (UPR), induced during periods of cellular and ER stress, aims to reduce unfolded protein load, and restore protein homeostasis by translational repression. ER stress can be the result of numerous conditions, including amino acid deprivation, viral replication and the presence of unfolded proteins, resulting in activation of the UPR. The UPR has three pathways activated through kinases, (1) protein kinase RNA (PKR)-like ER kinase (PERK), (2) inositol-requiring enzyme 1 (IRE1), and (3) activating transcription factor 6 (ATF6; Halliday and Mallucci, 2015). These kinases are kept in their inactive state by the binding immunoglobulin protein (BiP), during ER stress this protein binds to exposed hydrophobic domains of unfolded proteins and thereby allowing activation of these factors (Gething, 1999). In neurodegenerative diseases markers of the UPR, like PERK-P and eIF2α-P, have been reported in the brain of patients with neurodegenerative disease and in mouse models of neurodegeneration (Hetz and Mollereau, 2014; Scheper and Hoozemans, 2015).

### Protein compartmentalization

In the cell, misfolded proteins can be sequestered in distinct protein quality control compartments by chaperones and sorting factors. These compartments function as temporary storage until the protein can be refolded or degraded by the proteasome. Different compartments have been described in the literature that sequester different kind of misfolded proteins at various conditions, these include JUNQ, IPOD, Q-body, and aggresome (Sontag et al., 2014). Insoluble proteins are sequestered into insoluble protein deposit (IPOD) compartments that are located near the periphery of the cell (Kaganovich et al., 2008; Specht et al., 2011). If the proteasome is impaired these insoluble proteins can also be sequestered in aggresomes (Johnston et al., 1998), whereas, soluble misfolded proteins can be sequestered into ER-anchored structures named Q-bodies (Escusa-Toret et al., 2013). However, when the proteasome is impaired soluble ubiquitinated misfolded proteins are sequestered into ER-associated juxtanuclear quality control compartments (JUNQ) compartments (Kaganovich et al., 2008; Specht et al., 2011).

The JUNQ and Q-bodies concentrate misfolded proteins in distinct compartments together with chaperones and clearance factors, which makes processing them easier and more efficient. The IPOD and aggresomes are thought to protect the cell from toxic misfolded species, they do however also contain some disaggregases and autophagy related proteins and might therefore be recovered from these compartments (Kaganovich et al., 2008; Specht et al., 2011).

### Drivers of amyloid formation

Most studies on neurodegenerative diseases focus on either the toxic mechanisms or on the PQC system as possible targets for treatment. Only a few studies so far have focused directly on modifiers of the protein aggregation pathway. One example is the study that focused on a reduced insulin/insulin-like growth factor 1 signaling (IIS), which induces the assembly of Aβ into densely packed and larger fibrillar structures (Cohen et al., 2009). The exact mechanisms behind the formation of these tightly packed amyloid structures by IIS signaling remains to be unraveled.

MOAG-4 (modifier of aggregation 4) was found in a forward genetic screen using *C. elegans* models for neurodegenerative diseases, as an enhancer of aggregation and toxicity of several aggregation-prone disease proteins, including polyQ, α-synuclein, and Aβ (van Ham et al., 2010). MOAG-4 is a small protein of unknown function that is evolutionarily highly conserved. It contains a 4F5 domain of unknown function and is predicted to have a helix-loop-helix secondary structure. MOAG-4 itself was excluded from the polyQ aggregates in the *C. elegans* model. Based on biochemical experiments with worm extracts, MOAG-4 has been suggested to act on the formation of a compact aggregation intermediate. Furthermore, *in vitro* studies with mutant HTT exon 1 and MOAG-4 show a direct increase in aggregation (Unpublished data). Moreover, it was shown that the effect on aggregation works independent from DAF-16, HSF-1, and chaperones.

The human orthologs of MOAG-4 were found to be a two small proteins with unknown function, i.e., Small EDKR Rich Factor (SERF) 1A and 2. These two orhologs are 40% identical and 54% similar to MOAG-4 (van Ham et al., 2010). It was found that SERF1a (Falsone et al., 2012) is able to directly drive the amyloid formation of mutant HTT exon 1 and alpha-synuclein in an *in vitro* assay. It has been suggested that SERF1a directly affects the amyloidogenesis of alpha-synuclein by catalyzing the transition of an alpha-synuclein monomer into a amyloid-nucleating species (Falsone et al., 2012). From cell culture experiments we know that overexpression of SERF1a or SERF2, together with mutant HTT exon 1 results in an increase in toxicity and aggregation of the polyQ protein. Whereas, knock down of SERF using siRNA results in reduced toxicity and aggregation (van Ham et al., 2010).

## Protein homeostasis in aging

**Figure 1:**
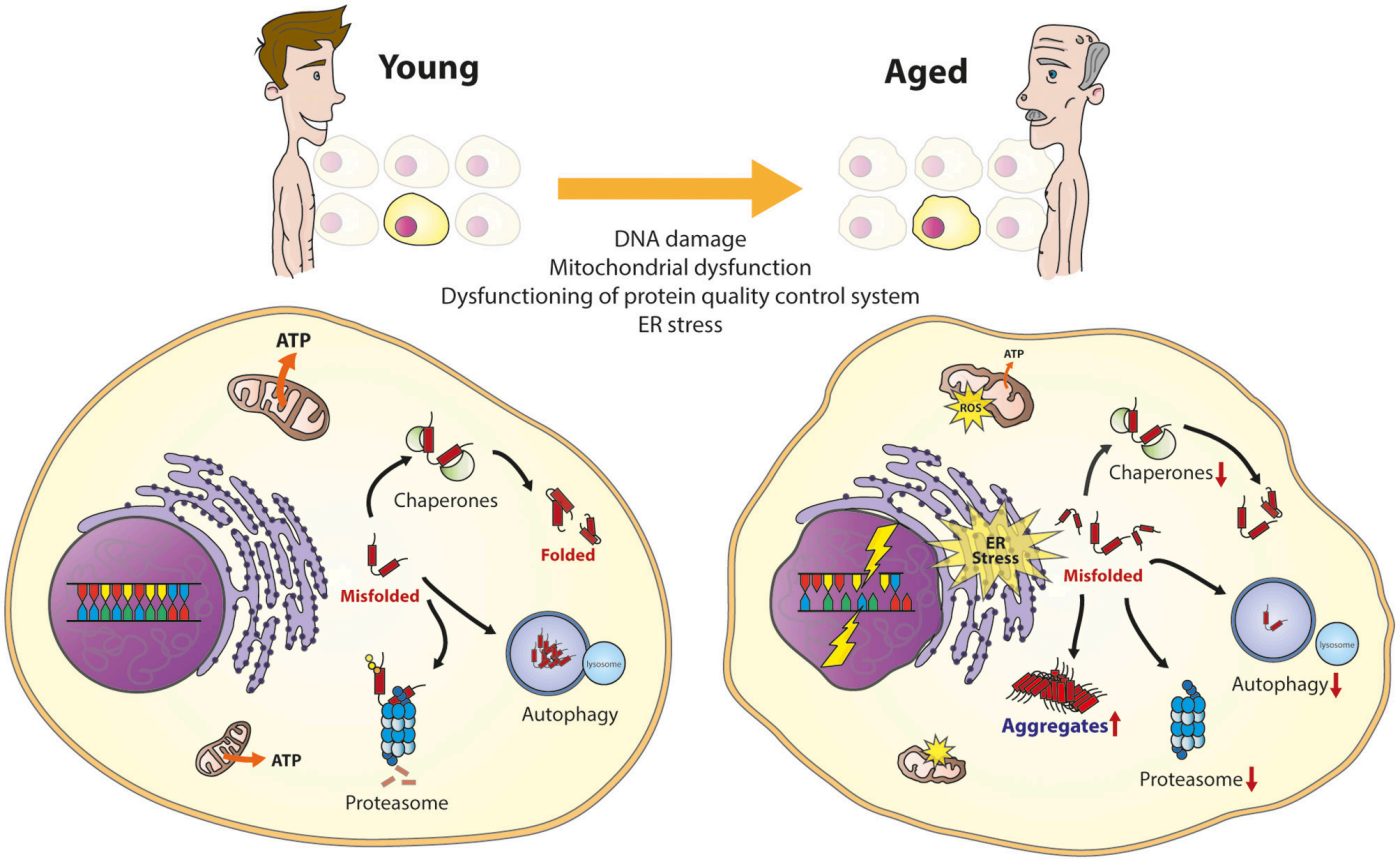
**The aging cell**. Important cellular processes are affected during aging. This will result in several cellular phenotypes, including the overload of the protein quality control system, DNA damage, mitochondrial dysfunction, and ER stress, together resulting in vulnerability to cell death.

Under normal conditions, the PQC can rapidly sense and correct cellular disturbances, by e.g., activating stress-induced cellular responses to restore the protein balance. During aging or when stress becomes chronic, the cell is challenged to maintain proper protein homeostasis (Figure 1; Koga et al., 2011; Labbadia and Morimoto, 2015; Radwan et al., 2017). Eventually, this can lead to chronic expression of misfolded and damaged proteins in the cell that can result in the formation of protein aggregates. The presence of aggregation-prone proteins contributes to the development of age-related diseases (Chiti and Dobson, 2006; Kakkar et al., 2014). The decline of protein homeostasis during aging is a complex phenomenon that involves a combination of different factors.

In line with the decreased protein homeostasis, there appears to be an impairment of the upregulation of molecular chaperones during aging (reviewed in Koga et al., 2011). This has been reported for HSP70 in senescent fibroblasts and in different tissues from different species, including monkeys (Fargnoli et al., 1990; Pahlavani et al., 1995; Hall et al., 2000). The importance to regulate the expression of HSPs is seen in flies and worms, where upregulation of HSPs leads to increase in lifespan (Walker et al., 2001; Hsu et al., 2003; Morley and Morimoto, 2003). Furthermore, lymphocytes from human centenarians show chaperone-preserved upregulation during heat shock (Ambra et al., 2004). It has been proposed that inability of the transcription factor HSF-1 to bind the chaperone gene promoter could explain the failure of *hsp70* to respond to stress during aging (Ambra et al., 2004; Singh et al., 2006). The functional decline of chaperones during aging also impairs proper folding of proteins in the ER resulting in activation of the UPR (reviwed in Taylor, 2016). Moreover, it has been shown that the capacity of some elements of the UPR, like PERK or IRE-1 also decline with age (Paz Gavilán et al., 2006; Taylor and Dillin, 2013).

Since all major classes of molecular chaperones, with the exception of the small HPSs, are ATPases it has been suggested that the depletion of ATP levels during aging due to mitochondria dysfunction would affect their activity (Kaushik and Cuervo, 2015; Yerbury et al., 2016). This is reflected by the repression of ATP-dependent chaperones and the induction of ATP-independent chaperones in the aging human brain (Brehme et al., 2014). This may contribute to the decline of chaperoning function during aging.

The activity of the degradation pathways of the PQC, autophagy and the proteasome, are also reduced during aging (reviewed in Koga et al., 2011 and Kaushik and Cuervo, 2015). The proteasome decline is caused by a down-regulation or deregulation of different proteasomal subunits and regulatory factors (Keller et al., 2000; Ferrington et al., 2005). In autophagy, fusion between the vesicles carrying the cytosolic cargo and lysosomal compartments is severely impaired. The chaperone-mediated autophagy is reduced due to progressively lower levels of receptors at the lysosomal membrane with age (Cuervo and Dice, 2000; Koga et al., 2011). Furthermore, a more active proteasome has been found in fibroblasts from centenarians (Chondrogianni et al., 2000; Koga et al., 2011) and reactivation of the proteasome and/or autophagy pathways increases lifespan of yeast, worms, and flies (Chondrogianni et al., 2015; Kaushik and Cuervo, 2015; Madeo et al., 2015). Altogether, showing the importance to remain a functioning PQC during aging.

## Mechanisms of protein toxicity in neurodegenerative diseases

Neuronal loss is one of the hallmarks of neurodegenerative diseases, where the neurons that are vulnerable to disease pathology differ for each disease. Initially it was thought that the protein aggregates that are observed in post-mortem brain material of patients were toxic (Davies et al., 1997; Kim et al., 1999). But this view shifted toward the hypothesis that the protein aggregates may actually be neuroprotective and that intermediate species are toxic. Indeed, the presence of diffuse protein resulted in higher toxicity compared to the presence of protein aggregates only (Arrasate et al., 2004). Furthermore, overexpression of HSF-1 in a cell model for HD leads to fewer but larger aggregates and increased viability (Pierce et al., 2010). The toxicity of intermediate species may arise from the presence of hydrophobic groups on their surface, that under normal physiological conditions would not be accessible within the cellular environment (Campioni et al., 2010). Accessible hydrophobicity in proteins can result in inappropriate interactions with many functional cellular components like the plasma membrane (Bucciantini et al., 2012). Therefore, aggregation might be a mechanism to assist in the clearance of misfolded proteins. In this regard, it has been described that chaperones can supress the toxicity of the oligomeric intermediate species by promoting the formation of larger aggregates (Lindberg et al., 2015). The question remains why these intermediate species are toxic. Different mechanisms have been suggested.

**Figure 2:**
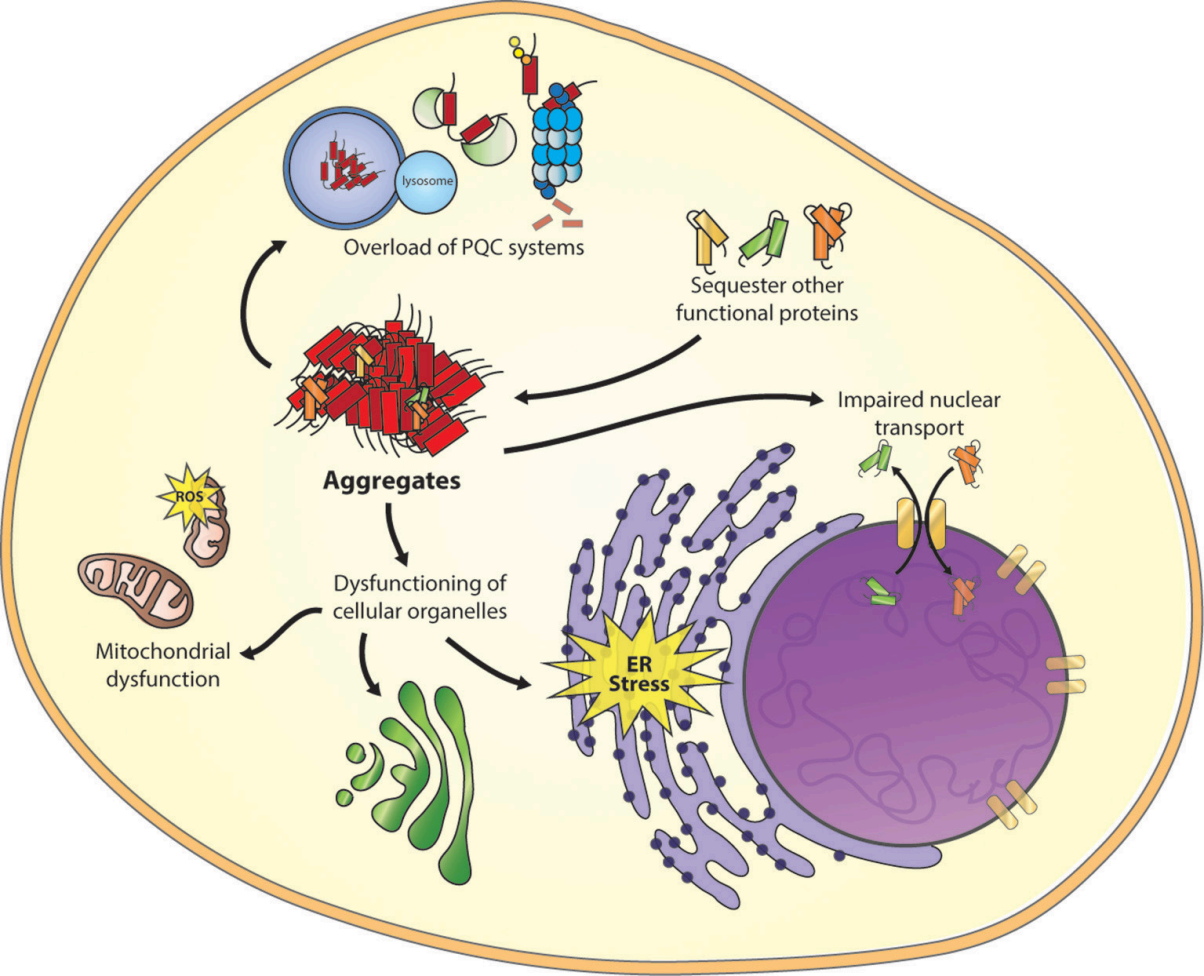
**Toxic mechanism of misfolded proteins**. Important cellular processes are affected as a result of misfolded proteins, including overload of the protein quality control (PQC) system, sequestering of functional proteins, disruption of the nuclear core complex and dysfunction of other cellular organelles as mitochondria, ER stress, and trans-Golgi network (the figure focuses on only one intermediate species, other species can be toxic too).

The increase of misfolded proteins during aging or disease can interfere with the PQC system by overloading the system (Figure 2), which in turn, can result in a propagation of folding defects and eventually protein aggregation (Labbadia and Morimoto, 2015). In polyQ worm models disruption of the PQC system by the polyQ aggregates resulted in the loss of function of several metastable proteins with destabilizing temperature-sensitive mutations, which also enhanced the aggregation of polyQ proteins (Gidalevitz et al., 2006). Furthermore, polyQ aggregates also impair the ubiquitin-proteasome system in cellular models for disease (Bence et al., 2001).

A “gain of function” mechanism is another form of cellular toxicity. Due to misfolding, hydrophobic residues of the protein can be located at the surface, permitting uncommon interactions with a wide range of cellular targets (Figure 2; Stefani and Dobson, 2003), including molecular chaperones (Park et al., 2013). Using cytotoxic artificial β-sheet protein aggregates it was found that the endogenous proteins that are sequestered by these aggregates share many physicochemical properties, including their relatively large size and enriched unstructured regions. Many of these proteins play essential roles in the several pathways, including translation, chromatin structure, and cytoskeleton. A loss of these proteins might results in a collapse of essential cellular functions and consequently may induce toxicity (Olzscha et al., 2011).

Recently, an effect of protein aggregation on the nuclear pore complex (NPC) was described. The GGGGCC (G_4_C_2_)repeat expansion in the non-coding region of the C9orf72 protein is the most common cause of sporadic and familial forms of amyotrophic lateral sclerosis (ALS) and frontal temporal dementia (FTD), (DeJesus-Hernandez et al., 2011; Renton et al., 2011). However, the exact mechanism of how the C9orf72 mutations contribute to the disease remains elusive. Two hypotheses are proposed, the first describes that the repeat containing transcripts can form intra-nuclear RNA foci that sequester various RNA-binding proteins (Donnelly et al., 2013), and the second describes the production of toxic dipeptide repeat proteins (DPRs; Ash et al., 2013). New insights have shown that mutant C9orf72 RNA affects nuclear transport of proteins and RNA (Figure 2). Loss of NPC proteins were found to enhance G_4_C_2_ repeat toxicity in fly and human cell models for disease (Freibaum et al., 2015; Zhang et al., 2015). Moreover, a screen to identify modifiers of toxicity by PR_50_DPR identified an enrichment in nucleocytoplasmic transport proteins, in which the six strongest hits were members of the karyopherin family of nuclear-import proteins (Jovičić et al., 2015). Furthermore, it was shown that nuclear localization of artificial β-sheet-, HTT-, and TDP-43 aggregates reduces toxicity in comparison to cytoplasmic aggregates. Because the cytoplasmic aggregates interfere with both import and export of proteins through the nuclear pore complex, they specifically affect proteins containing disordered and low complexity domains including many nuclear transport factors (Woerner et al., 2016). These studies show that reduced nuclear transport, as a result of protein aggregates, results in cellular toxicity. However, a better understanding of the exact mechanism behind these observations could provide us with a new therapeutic target to restore nuclear transport. In addition, several studies described toxic effects of protein aggregates on the functioning of other cellular organelles as the ER (Duennwald and Lindquist, 2008), mitochondrion (Rhein et al., 2009), and the trans-Golgi network (Cooper et al., 2006). Identifying different toxic consequences of misfolded proteins gives possibilities for treatments options.

Another mechanism of toxicity has been proposed in the literature, in which oligomeric aggregation intermediates bind and disrupt lipid membranes (Lashuel and Lansbury, 2006). Annular oligomeric structures have been identified for different amyloidogenic proteins, such as Aβ (Lashuel et al., 2002a,b), α-synuclein (Lashuel et al., 2002b,c), PrP (Sokolowski et al., 2003), or polyQ proteins (Wacker et al., 2004). These are pore-like structures that can embed into lipid bilayers and permeabilize membranes allowing the transit of small molecules. Diseases-associated mutations in Aβ (E22G) and α-synuclein (A53T and A30P) promote the formation of amyloid pores (Lashuel et al., 2002b,c; Lashuel and Lansbury, 2006). This is known as the amyloid pore hypothesis (Lashuel and Lansbury, 2006; Stöckl et al., 2013). Alternatively, a different explanation has been proposed for the permeabilization of membranes by α-synuclein, in which oligomers of this protein would not form pores, but they rather decrease the lipid order by incorporating between the tightly packed lipids, facilitating the diffusion of molecules through the membranes (Stöckl et al., 2013). Whether this alternative hypothesis can also be applicable to other amyloidgenic proteins still needs to be revealed. Furthermore, recent studies on non-pathological (Oropesa-Nuñez et al., 2016) and pathological proteins (Di Pasquale et al., 2010; Fukunaga et al., 2012; Mahul-Mellier et al., 2015) show that negatively charged ganglioside rich lipid rafts mediate toxicity of the prefibrillar oligomers.

Probably the toxicity of the disease proteins cannot be wholly explained by one of these mechanisms but rather by a combination of them.

### Gliosis

Neuroinflammation or gliosis, a reactive change of the glial cells in response to damage, is a common pathological feature in neurodegenerative diseases like AD and HD (Perry et al., 2010). However, whether inflammation plays an active or consequential role in disease is still a topic for debate. Glial cells are divided into two major classes: microglia and macroglia, where microglia are the phagocytes that are ubiquitously distributed in the brain and are mobilized after injury, disease, or infection. Pathological triggers, such as neuronal death or protein aggregates, activate the migration of microglia, which accumulate at the site of injury. This migration and recruitment is followed by the initiation of an innate immune response, which is a non-specific reaction resulting in the release of pro-inflammatory chemo- and cytokines (Gordon and Taylor, 2005; Hanisch and Kettenmann, 2007; Perry et al., 2010). The importance of glial cells in neurodegeneration is supported by the association found in genome wide association studies of immune receptors like TREM2 (Guerreiro et al., 2013; Jonsson et al., 2013) and CD33 (Griciuc et al., 2013) in AD. Gliosis has also been described for other neurodegenerative diseases as PD (Gerhard et al., 2006) and HD (Shin et al., 2005), but as the main aggregates are intracellular the response from microglia is not as strong as in AD.

### Spreading

Prion diseases (PrD) are a group of fatal neurodegenerative disorders caused by infectious proteins called prions. In humans most PrD can be identified under the name Creutzfeldt-Jakob disease (CJD), and in animals under the name bovine spongiform encephalopathy (BSE; Collinge, 2001). In PrD, the cellular form of the prion protein (PrP^C^) undergoes a conformational conversion into a β-sheet enriched isoform denoted as PrP^Sc^. This occurs when the PrP^Sc^ comes in contact with the mostly α-helical PrP^C^, as a result the PrP^C^ is misfolded into pathogenic PrP^Sc^, which in turn can become a template for conversion of other PrP^C^. The PrP^Sc^ form can form protein aggregates, prion deposits, often present as amyloid structures, which can propagate and possibly cause cell death (Collinge and Clarke, 2007; Collinge, 2016). PrDs are well-known to be able to spread throughout the brain via infectious prions. By the conversion of the protein into “seeds” due to stress, mutations or when PrP^C^ comes in contact with PrP^Sc^, it incites a chain reaction of PrP misfolding (Halliday et al., 2014). Prions are out of scope for this review, although they are one of the most relevant topics in neurodegenerative diseases especially due to their infectivity. This “prion-like” character of other neurodegenerative disease proteins has been proposed.

Spreading of Aβ in AD was first observed in a marmoset injected with brain extract from AD patients or AD affected marmosets, leading to AD pathology 6–10 years after injection (Baker et al., 1993; Ridley et al., 2006). Injection with only cerebrospinal fluid of AD patients or synthetic Aβ did not result in AD pathology in the marmoset (Ridley et al., 2006). As studies with marmosets are limited, these studies were replicated in mice to further investigate the spreading of Aβ. Brain extracts from AD patients or transgenic mouse models can initiate AD pathology in the brains of transgenic mice overexpressing the Swedish-mutated human APP (Meyer-Luehmann et al., 2006). Injection of synthetic human Aβ fibrils can induce AD pathology in mice, however the potency is lower than with AD brain extract (Stöhr et al., 2012). In mice depleted of amyloid-beta precursor protein (APP) there is no spreading of the disease, however if you take brain extracts of APP depleted mice inoculated with Aβ seeds, this can lead to propagation after second transmission for up to 180 days, suggesting extreme longevity of the Aβ “seeds” (Ye et al., 2015). Infectiousness of AD in humans has not yet been proven, though possible spreading of Aβ in humans was observed in two individual studies. The first study described four individuals with infectious Creutzfeldt-Jakob disease (CJD) who also showed moderate to severe AD pathology, they were injected as children with human growth hormone from cadaveric pituitary glands that contained PrP (Jaunmuktane et al., 2015). Another study observed infectious CJD in patients who received a dura mater transplant as a result of brain trauma or tumor, in five patients AD pathology was observed (Frontzek et al., 2016). As the patients in both studies did not carry pathogenic AD mutations or risk alleles and were too young to develop sporadic AD, the studies suggested that the treatment samples contained Aβ peptides.

Spreading of the PD pathology was first suggested when healthy dopaminergic neurons injected into the brain of PD patients showed Lewy body formation 11–16 years after transplantation (Kordower et al., 2008; Li et al., 2008). Follow-up studies in PD mouse models show that injection of brain extracts of PD transgenic mice results in the formation of PD pathology and increased mortality (Luk et al., 2012b; Mougenot et al., 2012). Furthermore, injection of synthetic α-synuclein (Luk et al., 2012a) or dementia with Lewy bodies (DLB) patient brain extract (Masuda-Suzukake et al., 2013) also results in PD pathology and neuronal death in healthy mice.

## Protein toxicity in non-neurodegenerative diseases

**Figure 3:**
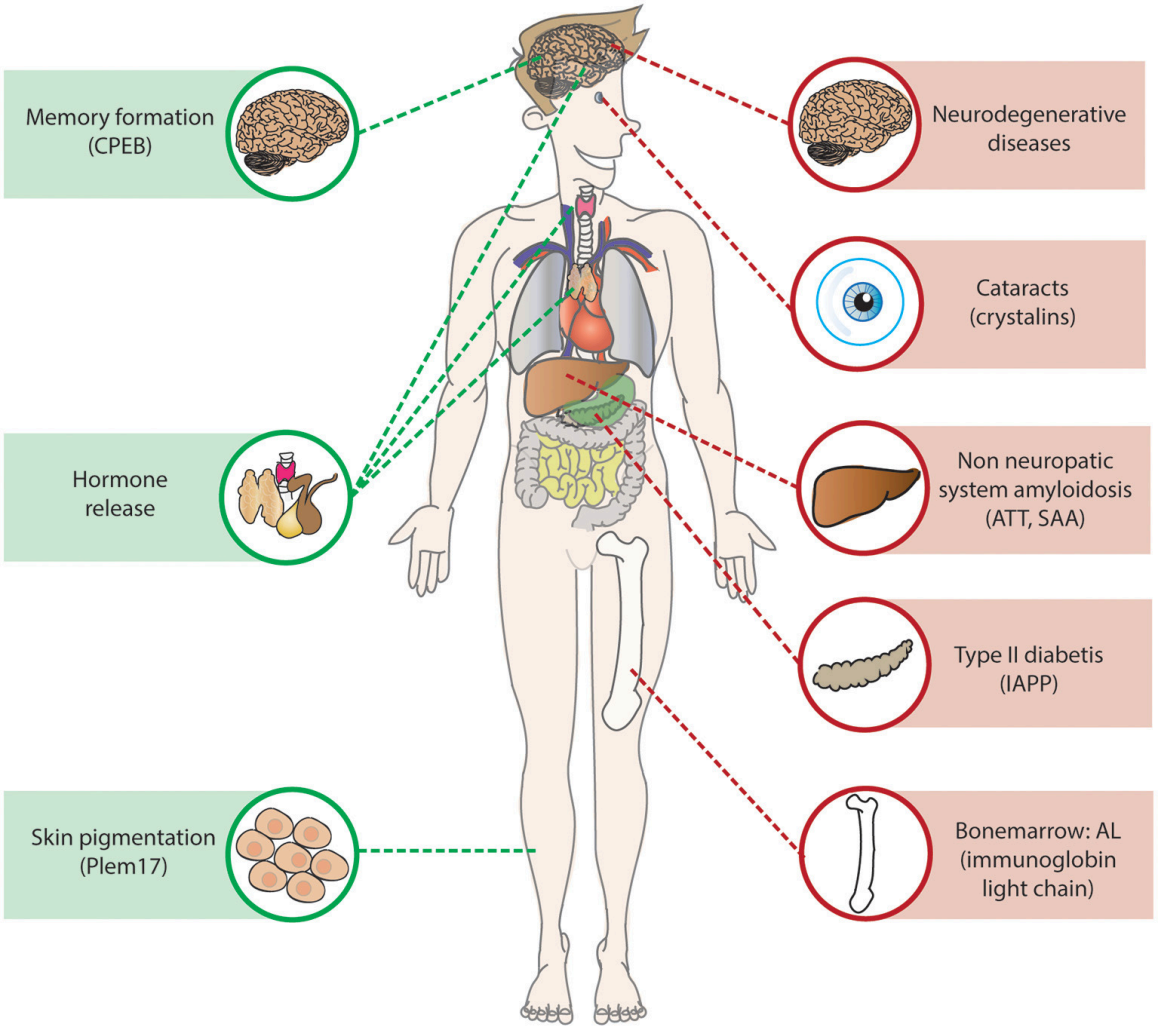
**Amyloids in health and disease**. Amyloids are present throughout the body in health and diseases, in green examples of functional amyloids described in the section is called “Functional Amyloid”. In red examples of amyloids resulted causing disease, the non-neuropathic systemic amyloidosis AL, ATT, and SAA are located at the point where they are produced, they do however affect multiple organs as the heart and kidney.

Protein aggregation is also involved in non-neurodegenerative diseases, and can be distinguish into two groups: non-neuropathic systemic amyloidosis and non-neuropathic localized disease (reviewed in Chiti and Dobson, 2006; Figure 3). Similar to neurodegenerative diseases they arise from the failure of a specific protein or peptide to acquire its native functional conformational state resulting in aggregation of the protein.

In non-neuropathic localized disease, the protein aggregation occurs in a single cell type or tissue other than the brain. The most well-known disease is type II diabetes, an age-related disease in which the glucose homeostasis is disturbed due to pancreatic islet β-cell dysfunction and death caused by aggregation of the islet amyloid polypeptide (IAPP; Abedini and Schmidt, 2013; Westermark and Westermark, 2013; Knowles et al., 2014). The amyloid deposits in the islet β-cells were first described over 100 years ago (Opie, 1901), and are a common feature in the pancreas of post-mortem material of type II diabetes patients. Pancreatic β-cells normally secrete insulin to regulate glucose uptake and metabolism in the body, mature IAPP is stored in the insulin secretory granule and co-secreted with insulin (Marzban et al., 2005). The exact role of IAPP is still unknown, although many functions have been suggested including regulation of glucose homeostasis (Abedini and Schmidt, 2013). The human IAPP is extremely amyloidogenic *in vitro*, and amyloids accumulate in the pancreatic islet in the majority of the type II diabetes patients (Westermark et al., 1989; Betsholtz et al., 1990).

Another common non-neuropathic localized disease is cataracts, a common form of blindness affecting more than 50% of the individuals over the age of 70. Normally, the lens can stay transparent throughout life, as there is no protein turnover or synthesis. In cataracts soluble proteins of the lens accumulate into amyloids, resulting in reduced transparency and thus reduced sight. Thirty percent of the lens is made up of the molecular chaperones αA-crystallin and αB-crystallin that maintain the solubility of other lens proteins. However, during aging damaged proteins accumulate which can lead to aggregation of the crystalline proteins (Bloemendal et al., 2004). Furthermore, the R120G mutation in αB-crystallin causes early onset cataracts (Vicart et al., 1998; Perng et al., 1999).

The non-neuropathic systemic amyloidosis are rare diseases caused by protein aggregation in multiple tissues (Falk et al., 1997). The most common non-neuropathic systemic amyloidosis is AL amyloidosis, a mainly sporadic disease that is characterized by aggregation of fragments of the misfolded monoclonal immunoglobin light chains in various organs (Comenzo, 2006; Chaulagain and Comenzo, 2013). The fragment can form β-sheets that are prone to form amyloids. The protein is produced by a plasma cell clone in the bone marrow and after internalization it can cause severe organ dysfunction and failure. The main organs affected by AL amyloidosis are the heart and kidneys, however, also other organs such as the liver, nervous system, and spleen can be affected (Falk et al., 1997; Comenzo, 2006). The treatment of the disease is aimed at eliminating the plasma cell clone, but a delay in the diagnosis of the disease often results in irreversible organ damage and thus poor prognoses (Chaulagain and Comenzo, 2013). Two other common non-neuropathic systemic amyloidosis are caused by transthyretin amyloidosis (ATTR) and serum amyloid A protein (SAA), both proteins are produced in the liver and affect various organs, however in ATTR heart failure is most common whereas SAA often results in renal failure (reviewed in Chiti and Dobson, 2006).

## Structural and functional properties of amyloid

**Figure 4:**
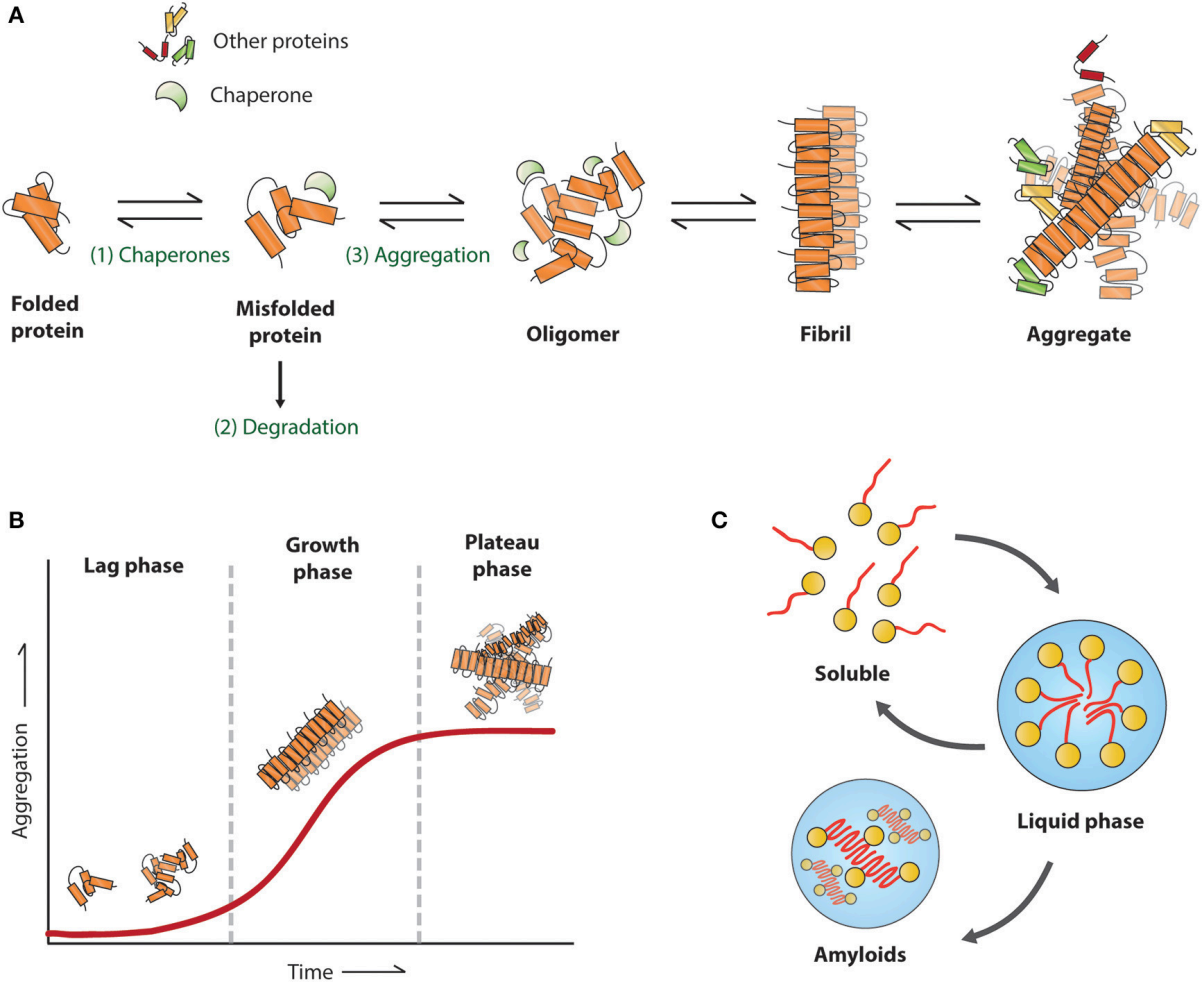
**Proposed mechanism for amyloid formation. (A)** A misfolded protein can be refolded (1), degraded (2), or aggregated (3), the first step in the aggregation pathway involves oligomers, followed by fibril formation around the fibril axis until the initial aggregates. **(B)** Schematic view of an *in vitro* assay with the corresponding aggregation stages for each phase **(C)** formation of liquid droplets.

The first amyloid was observed and described in 1854 by Rudolph Virchow for systemic amyloidosis (Sipe and Cohen, 2000). Since then, many diseases have been associated with amyloids. The proteins associated with protein aggregation diseases have no obvious similarity in sequences, native structures, or function. They do however, share characteristics in their amyloid state as they can undergo structural rearrangements leading to the formation of amyloid fibrils (Figure 4A). The amyloid fibrils have a highly organized and stable structure composed of proteins with a cross β-sheet structure oriented vertically to the fibril axis. They appear under the electron microscope as unbranched filamentous structures of just a few nanometers in diameter while up to micrometers in length. The cross β-sheet structure of amyloid fibrils provides a stable structure for the formation of continuous arrangement of hydrogen bonds between fibrils, eventually resulting in the formation of amyloids. The amyloid structures can be characterized by their following properties: insolubility to detergents like SDS and NP40, binding to specific dyes such as Thioflavins and Congo Red and resistance to proteases (reviewed in Chiti and Dobson, 2006). To learn more about intermediate species of the aggregation process the kinetics of aggregation can be studied *in vitro*. Using purified protein and a amyloid dye in a test tube, three phases of aggregation can be distinguished (Figure 4B). During the first lag phase there are mainly protein monomers and oligomers, this is followed by a rapid growth phase in which protein fibrils are formed, followed by a plateau phase in which the reaction is ended due to depletion of soluble proteins (Blanco et al., 2012).

The aggregation propensity of a protein is determined by short aggregation prone regions (APR) that are generally buried in the hydrophobic core of the protein. However, due to misfolding or mutations, these regions can be exposed and therefore self-assemble into aggregates. APRs are typically short sequence segments between 5 and 15 amino acids with high hydrophobicity, low net charge, and have a high tendency to form β-sheet structures (Ventura et al., 2004; Esteras-Chopo et al., 2005). Different algorithms have been generated to predict protein aggregation propensity of proteins or the effect of disease mutations, for example WALTZ an algorithm to determine amyloid forming sequences (Maurer-Stroh et al., 2010) and TANGO an algorithm that identifies the β-sheet regions of a protein sequence (Fernandez-Escamilla et al., 2004). Disease associated variants, not only related with neurodegenerative diseases, but also for cancers and immune disorders, tend to increase the predicted aggregation propensity of proteins (De Baets et al., 2015).

### Amyloid in disease

Proteins or peptides of most neurodegenerative diseases are intrinsically disordered in their free soluble form, like the Aβ peptide in AD and α-synclein in PD (Chiti and Dobson, 2006, 2009). Mutations in these disease proteins can make the protein even more prone to aggregate. For example, the A53T and A30P mutation of α-synclein found in early onset PD, promotes the acceleration of amyloid fibrils *in vitro* (Conway et al., 1998, 2000).

Furthermore, having too many copies of an aggregation-prone protein itself can lead to disease by increasing protein concentrations in the cell (Chiti and Dobson, 2006, 2009). This increase in protein concentration can switch the stability of the soluble state toward the amyloid state. For examples trisomy 21 patients (Down's syndrome) who have an extra copy of the APP protein and a highly increased risk of developing early onset AD (Wiseman et al., 2015). In addition, duplication or triplication of the α-synuclein gene (*SNCA*) results in early onset PD (Singleton et al., 2003), besides, the onset, progression, and severity of the disease phenotype increases with the number of copies of the *SNCA* gene (Chartier-Harlin et al., 2004). To this end, also proteins that regulate expression levels of disease proteins can cause or influence diseases, an example is the RNA binding protein Pumilio1 that regulates the mRNA levels of *Ataxin1* RNA. Pumilio1 haploinsufficiency accelerates the SCA1 disease progression in a mouse model for disease due to increase of the Atxn1 mRNA and protein levels (Gennarino et al., 2015). If protein levels strongly influence the toxicity and disease phenotype this would suggests that lowering the protein load could be a therapeutic strategy. This was shown in an AD mouse model where the APP transgenes could be turned off with a tet-off system, when the APP levels were halted there was an arrest of the AD pathology without clearance of the excising plaques (Jankowsky et al., 2005), resulting in a significant effect on cognitive function (Fowler et al., 2014). Indicating that the concentration of disease proteins influences the disease progression, thereby affecting the development of disease.

That structural differences between amyloid “strains” can influence disease phenotype was first described for PrD, where isolated strains of PrP aggregates from different sources propagated different in mice showing distinct incubation times and patterns of neuropathology (Fraser and Dickinson, 1973). Furthermore, different human PrP strains have been associated with differences in proteinase K digestion and distinct phenotypes of neuropathology (reviewed in Collinge and Clarke, 2007). More recently, investigation of two familial human AD patients with different disease symptoms, showed a structural difference in amyloid fibril structure (Lu et al., 2013). Furthermore, Arctic and Swedisch familial AD patients brain homogenate results in distinct disease phenotypes in transgenic mice even after serial passage (Watts et al., 2014). Comparable results were found for Tau, another aggregation-prone protein involved in AD. Injection of two distinct *in vitro* generated Tau strains into transgenic mice resulted in distinct pathologies up to three generations (Sanders et al., 2014). These studies suggest that variations in the properties of amyloid fibrils could affect disease pathology and symptoms. How these different strains are formed and how they contribute to the disease pathology is still unknown. It was however found that reduced IIS signaling in the APP/PS1 AD mouse model induces the assembly of Aβ into densely packed and larger fibrillar structures later in life, resulting in reduced AD symptoms (Cohen et al., 2009). Suggesting that altering the structure of the amyloid fibrils could be beneficial for patients, as certain structures appear to be more toxic than others.

### Functional amyloid

Amyloids structures are known to have biological functions in *Escherichia coli*, silkworms, fungi, and mammals (Fowler et al., 2007). One example in mammals is Pmel17 (Figure 3), a highly aggregation-prone protein that forms functional amyloid structures that are the main component of melanosome fibrils, membrane-bound organelles in pigment cells that store and synthesize melanin. Plem17 contains a partial repeat sequence that is essential for amyloid formation that can only be formed in the mildly acid pH of melanosomes (McGlinchey et al., 2009). The exact function of Pmel17 in melanosomes is unknown, although a role in protection against oxidative damage has been suggested, as well as a role in concentrating melanins to facilitate intra- and extracellular transport (Watt et al., 2013).

More functional amyloids in mammals can be found in hormone release, it was shown that certain hormones can be stored in amyloid-like aggregates in the secretory granules of the cell. These secretory granules have a β-sheet rich structure that is Thioflavin S and Congo Red positive and are able to release functional monomeric hormone structures upon dilution, and show only moderately toxicity on cell cultures, possibly due to their membrane-encapsulated state in the granules (Maji et al., 2009).

Interestingly, the formation of amyloids has recently been associated with long-term memory. The cytoplasmic polyadenylation element-binding protein (CPEBs) is a regulator of activity dependent synthesis in the synapse. The fly homolog Orb2 (Majumdar et al., 2012) and mouse homolog CPEB3 (Fioriti et al., 2015) are present in the brain as a monomer and SDS-resistant oligomer. Activation of the fly or mouse brain results in increase of the oligomeric Orb2/CPEB3 species. Selectively disrupting the oligomerization capacity of Orb2 by a genetic mutation resulted in long-term memory loss in flies (Majumdar et al., 2012) and loss of CPEB3 in the mouse brain resulted in impaired long term memory (Fioriti et al., 2015). Orb2 alters protein composition of the synapse by a mechanism in which the oligomeric Orb2 stimulates translation by elongation and protection of poly(A) tail, whereas the monomeric Orb2 does the contrary (Khan et al., 2015).

These functional amyloids point toward the origin of amyloid-prone sequences and their suppressors and enhancers. Even though, these functional amyloids have not been linked to human diseases, a functional role might be the case for the amyloid domains of disease proteins with unknown functions. More studies toward understanding the functionality of these amyloids and the difference with the disease amyloids are required to have a better understanding of why certain amyloids are toxic while others are not.

### Liquid droplets/liquid-to-solid-phase transition

It was recently found that proteins with prion-like domains can form functional non-membrane-bound organelles like ribonucleoprotein (RNP) bodies, that behave like liquid droplets which can rapidly assemble and disassemble in a response to changes in the cellular environment (Han et al., 2012; Kato et al., 2012). The RNP bodies include processing bodies and stress granules in the cytoplasm, and nucleoli, Cajal bodies and PML bodies in the nucleus. Due to the dynamic structures of RNPs there is free diffusion within the bodies and rapid exchange with the external environment. Like in liquid-liquid phase separation (LLPS) the RNP bodies exhibit liquid-like behaviors such as wetting, dripping, and relaxation to spherical structures upon fusion (Chong and Forman-Kay, 2016; Uversky, 2017). These properties can facilitate their function, by allowing for high concentration of molecular components that nonetheless remain dynamic within the droplet. Interestingly many of the proteins known to segregate into RNP bodies contain repetitive putatively prion-like domains, that can reversibly transform from soluble to a dynamic amyloid-like state (Kato et al., 2012). Furthermore, dysregulation of these RNP bodies by RNA-binding proteins have been associated with neurodegenerative diseases as ALS (Ramaswami et al., 2013).

The link for these RNP bodies in disease was first found for the FUS protein, mutations in the N-terminal prion-like domain have been associated with ALS, and FTD. This protein plays an important role in RNA processing and localizes to both cytoplasmic RNP bodies and transcriptionally active nuclear puncta, the prion-like domain is essential for forming these liquid-like compartments (Shelkovnikova et al., 2014). The N-terminus of FUS is structurally disordered both as a monomer and in its liquid state (Burke et al., 2015). *In vitro*, these droplets convert with time from a liquid to an aggregated state (Figure 4C), and this conversion is accelerated by patient-derived mutations (Patel et al., 2015). Furthermore, concentrated liquid droplets increase the probability of aggregation events of RNA-binding proteins in the RNP bodies in a concentration dependent manner (Molliex et al., 2015). mRNA itself can drive its phase transition of the disordered RNA binding-protein Whi3, and thereby altering the droplet viscosity, dynamics, and propensity to fuse. Suggesting that, mRNA contains biophysical properties of phase-separated compartments. Like FUS droplets the Whi3 droplets mature over time and appear to be fibrillar (Zhang et al., 2015).

This new line of research indicates another possible function for prion-like domains of various proteins and the proteins it interacts with. Furthermore, research to these RNP bodies shows possible reasons why these proteins form amyloids. However, much is still unknown about the exact mechanisms of the amyloid like domains and the RNP bodies that have to be investigated.

## Conclusion

Protein aggregation is a complex process influenced by many factors, pathways, and mechanisms. Under the right conditions any protein could form amyloid-like structures (Chiti and Dobson, 2006). Although amyloids have been traditionally related to diseases, they also have diverse functions in organisms from bacteria to human that may underlie their nature. Nevertheless, the toxicity of amyloid intermediate species associated with disease makes protein aggregation a process that has to be under tight control and regulation. In this context, aging is a key risk factor due to the progressive decline of protein homeostasis, which leads to increased protein misfolding and aggregation. This can eventually result in the onset of age-related diseases characterized by protein aggregation. Mutations or duplications that lead to the appearance of aggregation-prone proteins that are constitutively expressed in the cell, creating a chronic stress situation, leads to an early onset of those diseases due to the deregulation of the protein homeostasis balance.

As the human population becomes older, it is essential to understand the processes underlying age-related diseases that are the result of protein aggregation and its associated toxicity. This is a very broad research field, ranging from biophysics to clinical trials. Every year discoveries are made that involve the identification of factors affecting protein aggregation. Examples include the discovery of modifiers of protein aggregation such as MOAG-4/SERF, or the processes where protein aggregation and amyloid structure are involved, like RNA granules and liquid droplets formation. It can be concluded that the overall knowledge of the aggregation process is improving, which will allow for the development of new and accurate treatments against aggregation-linked diseases.

## Author contributions

ES wrote the review with the contribution and substantial intellectual input from MK, EN, and AM. MK did the figure design.

### Conflict of interest statement

The authors declare that the research was conducted in the absence of any commercial or financial relationships that could be construed as a potential conflict of interest.

## Abbreviations

Aβ: amyloid-beta
AD: Alzheimer disease
ALS: amyotrophic lateral sclerosis
APP: amyloid precursor protein
APR: aggregation prone region
ATTR: transthyretin amyloidosis
CMA: chaperone mediated autophagy
CJD: Creutzfeldt-Jakob disease
CPEB: cytoplasmic polyadenylation element-binding protein
DLB: dementia with Lewy bodies
ER: endoplasmic reticulum
FTD: frontal temporal dementia
HD: Huntington disease
HSF-1: heat shock factor 1
HSP: heat shock protein
HTT: huntingtin
IAPP: islet amyloid polypeptide
IIS: insulin/insulin-like growth factor 1 signaling
IPOD: insoluble protein deposit
JUNQ: juxtanuclear quality control compartments
LLPS: liquid-liquid phase separation
MOAG-4: modifier of aggregation 4
NPC: nuclear pore complex
PD: Parkinson disease
PolyQ: polyglutamine
PQC: protein quality control
PrD: prion disease
PrP: prion protein
RNP: ribonucleoprotein
SAA: serum amyloid protein
SERF: small EDKR rich factor
UPR: unfolded protein response
UPS: ubiquitin-proteasome system.

## References

[B1] Abedini A. Schmidt A. M. (2013). Mechanisms of islet amyloidosis toxicity in type 2 diabetes. FEBS Lett. 587, 1119–1127. 10.1016/j.febslet.2013.01.017 23337872 PMC4557799

[B2] Ambra R. Mocchegiani E. Giacconi R. Canali R. Rinna A. Malavolta M.. (2004). Characterization of the hsp70 response in lymphoblasts from aged and centenarian subjects and differential effects of *in vitro* zinc supplementation. Exp. Gerontol. 39, 1475–1484. 10.1016/j.exger.2004.07.009 15501017

[B3] Arrasate M. Mitra S. Schweitzer E. S. Segal M. R. Finkbeiner S. (2004). Inclusion body formation reduces levels of mutant huntingtin and the risk of neuronal death. Nature 431, 805–810. 10.1038/nature02998 15483602

[B4] Ash P. E. Bieniek K. F. Gendron T. F. Caulfield T. Lin W. L. DeJesus-Hernandez M.. (2013). Unconventional translation of C9ORF72 GGGGCC expansion generates insoluble polypeptides specific to c9FTD/ALS. Neuron 77, 639–646. 10.1016/j.neuron.2013.02.004 23415312 PMC3593233

[B5] Auluck P. K. Bonini N. M. (2002). Pharmacological prevention of Parkinson disease in Drosophila. Nat. Med. 8, 1185–1186. 10.1038/nm1102-1185 12411925

[B6] Baker H. F. Ridley R. M. Duchen L. W. Crow T. J. Bruton C. J. (1993). Evidence for the experimental transmission of cerebral beta-amyloidosis to primates. Int. J. Exp. Pathol. 74, 441–454. 8217779 PMC2002177

[B7] Bence N. F. Sampat R. M. Kopito R. R. (2001). Impairment of the ubiquitin-proteasome system by protein aggregation. Science 292, 1552–1555. 10.1126/science.292.5521.1552 11375494

[B8] Betsholtz C. Christmanson L. Engström U. Rorsman F. Jordan K. O'Brien T. D.. (1990). Structure of cat islet amyloid polypeptide and identification of amino acid residues of potential significance for islet amyloid formation. Diabetes 39, 118–122. 2210054 10.2337/diacare.39.1.118

[B9] Blanco L. P. Evans M. L. Smith D. R. Badtke M. P. Chapman M. R. (2012). Diversity, biogenesis and function of microbial amyloids. Trends Microbiol. 20, 66–73. 10.1016/j.tim.2011.11.005 22197327 PMC3278576

[B10] Bloemendal H. de Jong W. Jaenicke R. Lubsen N. H. Slingsby C. Tardieu A. (2004). Ageing and vision: structure, stability and function of lens crystallins. Prog. Biophys. Mol. Biol. 86, 407–485. 10.1016/j.pbiomolbio.2003.11.012 15302206

[B11] Bonifati V. Rizzu P. van Baren M. J. Schaap O. Breedveld G. J. Krieger E.. (2003). Mutations in the DJ-1 gene associated with autosomal recessive early-onset Parkinsonism. Science 299, 256–259. 10.1126/science.1077209 12446870

[B12] Brehme M. Voisine C. Rolland T. Wachi S. Soper J. H. Zhu Y.. (2014). A chaperome subnetwork safeguards proteostasis in aging and neurodegenerative disease. Cell Rep. 9, 1135–1150. 10.1016/j.celrep.2014.09.042 25437566 PMC4255334

[B13] Bucciantini M. Nosi D. Forzan M. Russo E. Calamai M. Pieri L.. (2012). Toxic effects of amyloid fibrils on cell membranes: the importance of ganglioside GM1. FASEB J. 26, 818–831. 10.1096/fj.11-189381 22071505

[B14] Burke K. A. Janke A. M. Rhine C. L. Fawzi N. L. (2015). Residue-by-residue view of *in vitro* FUS granules that bind the C-terminal domain of RNA polymerase II. Mol. Cell 60, 231–241. 10.1016/j.molcel.2015.09.006 26455390 PMC4609301

[B15] Campioni S. Mannini B. Zampagni M. Pensalfini A. Parrini C. Evangelisti E.. (2010). A causative link between the structure of aberrant protein oligomers and their toxicity. Nat. Chem. Biol. 6, 140–147. 10.1038/nchembio.283 20081829

[B16] Chartier-Harlin M. C. Kachergus J. Roumier C. Mouroux V. Douay X. Lincoln S.. (2004). α-synuclein locus duplication as a cause of familial Parkinson's disease. Lancet 364, 1167–1169. 10.1016/S0140-6736(04)17103-1 15451224

[B17] Chartier-Harlin M. C. Crawford F. Houlden H. Warren A. Hughes D. Fidani L.. (1991). Early-onset Alzheimer's disease caused by mutations at codon 717 of the beta-amyloid precursor protein gene. Nature 353, 844–846. 10.1038/353844a0 1944558

[B18] Chaulagain C. P. Comenzo R. L. (2013). New insights and modern treatment of AL amyloidosis. Curr. Hematol. Malig. Rep. 8, 291–298. 10.1007/s11899-013-0175-0 24026941

[B19] Chiti F. Dobson C. M. (2006). Protein misfolding, functional amyloid, and human disease. Annu. Rev. Biochem. 75, 333–366. 10.1146/annurev.biochem.75.101304.123901 16756495

[B20] Chiti F. Dobson C. M. (2009). Amyloid formation by globular proteins under native conditions. Nat. Chem. Biol. 5, 15–22. 10.1038/nchembio.131 19088715

[B21] Chondrogianni N. Georgila K. Kourtis N. Tavernarakis N. Gonos E. S. (2015). 20S proteasome activation promotes life span extension and resistance to proteotoxicity in Caenorhabditis elegans. FASEB J. 29, 611–622. 10.1096/fj.14-252189 25395451 PMC4314225

[B22] Chondrogianni N. Petropoulos I. Franceschi C. Friguet B. Gonos E. S. (2000). Fibroblast cultures from healthy centenarians have an active proteasome. Exp. Gerontol. 35, 721–728. 10.1016/S0531-5565(00)00137-6 11053662

[B23] Chong P. A. Forman-Kay J. D. (2016). Liquid–liquid phase separation in cellular signaling systems. Curr. Opin. Struct. Biol. 41, 180–186. 10.1016/j.sbi.2016.08.001 27552079

[B24] Ciechanover A. (2006). Intracellular protein degradation: from a vague idea thru the lysosome and the ubiquitin-proteosome system and onto human diseases and drug targeting. ASH Educ. B. 2006, 1–12. 10.1182/asheducation-2006.1.1 17124032

[B25] Ciechanover A. Kwon Y. T. (2015). Degradation of misfolded proteins in neurodegenerative diseases: therapeutic targets and strategies. Exp. Mol. Med. 47:e147. 10.1038/emm.2014.117 25766616 PMC4351408

[B26] Ciechanover A. Orian A. Schwartz A. L. (2000). Ubiquitin-mediated proteolysis: biological regulation via destruction. BioEssays 22, 442–451. 10.1002/(SICI)1521-1878(200005)22:5<442::AID-BIES6>3.0.CO;2-Q 10797484

[B27] Cohen E. Paulsson J. F. Blinder P. Burstyn-Cohen T. Du D. Estepa G.. (2009). Reduced IGF-1 signaling delays age-associated proteotoxicity in mice. Cell 139, 1157–1169. 10.1016/j.cell.2009.11.014 20005808 PMC3017511

[B28] Collinge J. (2001). Prion diseases of humans and animals : their causes and molecular basis. Annu. Rev. 24, 519–550. 10.1146/annurev.neuro.24.1.519 11283320

[B29] Collinge J. (2016). Mammalian prions and their wider relevance in neurodegenerative diseases. Nature 539, 217–226. 10.1038/nature20415 27830781

[B30] Collinge J. Clarke A. R. (2007). A general model of prion strains and their pathogenicity. Science 318, 930–936. 10.1126/science.1138718 17991853

[B31] Comenzo R. L. (2006). Current and emerging views and treatments of systemic immunoglobulin light-chain (AL) amyloidosis, in The Kidney in Plasma Cell Dyscrasias (Basel: KARGER), 195–210. 10.1159/000096768 17075231

[B32] Conway K. A. Harper J. D. Lansbury P. T. (2000). Fibrils formed *in vitro* from α-synuclein and two mutant forms linked to Parkinson's disease are typical amyloid. Biochemistry 39, 2552–2563. 10.1021/bi991447r 10704204

[B33] Conway K. A. Harper J. D. Lansbury P. T. (1998). Accelerated *in vitro* fibril formation by a mutant alpha-synuclein linked to early-onset Parkinson disease. Nat. Med. 4, 1318–1320. 10.1038/3311 9809558

[B34] Cooper A. A. Gitler A. D. Cashikar A. Haynes C. M. Hill K. J. Bhullar B.. (2006). Alpha-synuclein blocks ER-Golgi traffic and Rab1 rescues neuron loss in Parkinson's models. Science 313, 324–328. 10.1126/science.1129462 16794039 PMC1983366

[B35] Cuervo A. M. Dice J. F. (2000). Age-related decline in chaperone-mediated autophagy. J. Biol. Chem. 275, 31505–31513. 10.1074/jbc.M002102200 10806201

[B36] Davies S. W. Turmaine M. Cozens B. A. DiFiglia M. Sharp A. H. Ross C. A.. (1997). Formation of neuronal intranuclear inclusions underlies the neurological dysfunction in mice transgenic for the HD mutation. Cell 90, 537–548. 10.1016/S0092-8674(00)80513-9 9267033

[B37] De Baets G. Van Doorn L. Rousseau F. Schymkowitz J. (2015). Increased aggregation is more frequently associated to human disease-associated mutations than to neutral polymorphisms. PLoS Comput. Biol. 11:e1004374. 10.1371/journal.pcbi.1004374 26340370 PMC4560525

[B38] Dedmon M. M. Christodoulou J. Wilson M. R. Dobson C. M. (2005). Heat shock protein 70 inhibits alpha-synuclein fibril formation via preferential binding to prefibrillar species. J. Biol. Chem. 280, 14733–14740. 10.1074/jbc.M413024200 15671022

[B39] DeJesus-Hernandez M. Mackenzie I. R. Boeve B. F. Boxer A. L. Baker M. Rutherford N. J.. (2011). Expanded GGGGCC hexanucleotide repeat in noncoding region of C9ORF72 causes chromosome 9p-linked FTD and ALS. Neuron 72, 245–256. 10.1016/j.neuron.2011.09.011 21944778 PMC3202986

[B40] Di Pasquale E. Fantini J. Chahinian H. Maresca M. Taïeb N. Yahi N. (2010). Altered ion channel formation by the Parkinson's-disease-linked E46K mutant of α-synuclein is corrected by GM3 but not by GM1 gangliosides. J. Mol. Biol. 397, 202–218. 10.1016/j.jmb.2010.01.046 20114052

[B41] Donnelly C. J. Zhang P. W. Pham J. T. Heusler A. R. Mistry N. A. Vidensky S.. (2013). RNA toxicity from the ALS/FTD C9ORF72 expansion is mitigated by antisense intervention. Neuron 80, 415–428. 10.1016/j.neuron.2013.10.015 24139042 PMC4098943

[B42] Duennwald M. L. Lindquist S. (2008). Impaired ERAD and ER stress are early and specific events in polyglutamine toxicity. Genes Dev. 22, 3308–3319. 10.1101/gad.1673408 19015277 PMC2600758

[B43] Ellis J. (1987). Proteins as molecular chaperones. Nature 328, 378–379. 10.1038/328378a0 3112578

[B44] Ellis R. J. Hartl F. U. (1999). Principles of protein folding in the cellular environment. Curr. Opin. Struct. Biol. 9, 102–110. 10.1016/S0959-440X(99)80013-X 10047582

[B45] Escusa-Toret S. Vonk W. I. Frydman J. (2013). Spatial sequestration of misfolded proteins by a dynamic chaperone pathway enhances cellular fitness during stress. Nat. Cell Biol. 15, 1231–1243. 10.1038/ncb2838 24036477 PMC4121856

[B46] Esteras-Chopo A. Serrano L. López de la Paz M. (2005). The amyloid stretch hypothesis: recruiting proteins toward the dark side. Proc. Natl. Acad. Sci. U.S.A. 102, 16672–16677. 10.1073/pnas.0505905102 16263932 PMC1283810

[B47] Falk R. H. Comenzo R. L. Skinner M. (1997). The systemic amyloidoses. N.Engl. J. Med. 337, 898–909. 10.1056/NEJM199709253371306 9302305

[B48] Falsone S. F. Meyer N. H. Schrank E. Leitinger G. Pham C. L. Fodero-Tavoletti M. T.. (2012). SERF protein is a direct modifier of amyloid fiber assembly. Cell Rep. 2, 358–371. 10.1016/j.celrep.2012.06.012 22854022 PMC3807654

[B49] Fargnoli J. Kunisada T. Fornace A. J. Jr. Schneider E. L. Holbrook N. J. (1990). Decreased expression of heat shock protein 70 mRNA and protein after heat treatment in cells of aged rats. Proc. Natl. Acad. Sci. U.S.A. 87, 846–850. 10.1073/pnas.87.2.846 2300568 PMC53363

[B50] Fernandez-Escamilla A.-M. Rousseau F. Schymkowitz J. Serrano L. (2004). Prediction of sequence-dependent and mutational effects on the aggregation of peptides and proteins. Nat. Biotechnol. 22, 1302–1306. 10.1038/nbt1012 15361882

[B51] Ferrington D. A. Husom A. D. Thompson L. V. (2005). Altered proteasome structure, function, and oxidation in aged muscle. FASEB J. 19, 644–646. 10.1096/fj.04-2578fje 15677694

[B52] Fioriti L. Myers C. Huang Y. Y. Li X. Stephan J. S. Trifilieff P.. (2015). The persistence of hippocampal-based memory requires protein synthesis mediated by the prion-like protein CPEB3. Neuron 86, 1433–1448. 10.1016/j.neuron.2015.05.021 26074003

[B53] Flower T. R. Chesnokova L. S. Froelich C. A. Dixon C. Witt S. N. (2005). Heat shock prevents alpha-synuclein-induced apoptosis in a yeast model of Parkinson's disease. J. Mol. Biol. 351, 1081–1100. 10.1016/j.jmb.2005.06.060 16051265

[B54] Fowler D. M. Koulov A. V. Balch W. E. Kelly J. W. (2007). Functional amyloid – from bacteria to humans. Trends Biochem. Sci. 32, 217–224. 10.1016/j.tibs.2007.03.003 17412596

[B55] Fowler S. W. Chiang A. C. Savjani R. R. Larson M. E. Sherman M. A. Schuler D. R.. (2014). Genetic modulation of soluble Aβ rescues cognitive and synaptic impairment in a mouse model of Alzheimer's disease. J. Neurosci. 34, 7871–7885. 10.1523/JNEUROSCI.0572-14.2014 24899710 PMC4044248

[B56] Fraser H. Dickinson A. G. (1973). Scrapie in mice. Agent-strain differences in the distribution and intensity of grey *matter vacuolation*. J. Comp. Pathol. 83, 29–40. 10.1016/0021-9975(73)90024-8 4199908

[B57] Freibaum B. D. Lu Y. Lopez-Gonzalez R. Kim N. C. Almeida S. Lee K.-H.. (2015). GGGGCC repeat expansion in C0ORF72 compromises nucleocytoplasmic transport. Nature 525, 129–133. 10.1038/nature14974 26308899 PMC4631399

[B58] Frontzek K. Lutz M. I. Aguzzi A. Kovacs G. G. Budka H. (2016). Amyloid-β pathology and cerebral amyloid angiopathy are frequent in iatrogenic Creutzfeldt-Jakob disease after dural grafting. Swiss Med. Wkly. 146:w14287. 10.4414/smw.2016.14287 26812492

[B59] Fukunaga S. Ueno H. Yamaguchi T. Yano Y. Hoshino M. Matsuzaki K. (2012). GM1 cluster mediates formation of toxic Aβ fibrils by providing hydrophobic environments. Biochemistry 51, 8125–8131. 10.1021/bi300839u 23009396

[B60] Gao X. Carroni M. Nussbaum-Krammer C. Mogk A. Nillegoda N. B. Szlachcic A.. (2015). Human Hsp70 disaggregase reverses Parkinson's-linked α-synuclein amyloid fibrils. Mol. Cell 59, 781–793. 10.1016/j.molcel.2015.07.012 26300264 PMC5072489

[B61] Gennarino V. A. Singh R. K. White J. J. De Maio A. Han K. Kim J.-Y.. (2015). Pumilio1 haploinsufficiency leads to SCA1-like neurodegeneration by increasing wild-type Ataxin1 levels. Cell 160, 1087–1098. 10.1016/j.cell.2015.02.012 25768905 PMC4383046

[B62] Gerhard A. Pavese N. Hotton G. Turkheimer F. Es M. Hammers A. (2006). *In vivo* imaging of microglial activation with [11C](R)-PK11195 PET in idiopathic Parkinson's disease. Neurobiol. Dis. 21, 404–412. 10.1016/j.nbd.2005.08.002 16182554

[B63] Gething M. J. (1999). Role and regulation of the ER chaperone BiP. Semin. Cell Dev. Biol. 10, 465–472. 10.1006/scdb.1999.0318 10597629

[B64] Gidalevitz T. Ben-Zvi A. Ho K. H. Brignull H. R. Morimoto R. I. (2006). Progressive disruption of cellular protein folding in models of polyglutamine diseases. Science 311, 1471–1474. 10.1126/science.1124514 16469881

[B65] Gillis J. Schipper-Krom S. Juenemann K. Gruber A. Coolen S. van Den Nieuwendijk R.. (2013). The DNAJB6 and DNAJB8 protein chaperones prevent intracellular aggregation of polyglutamine peptides. J. Biol. Chem. 288, 17225–17237. 10.1074/jbc.m112.421685 23612975 PMC3682527

[B66] Glover J. R. Lindquist S. (1998). Hsp104, Hsp70, and Hsp40: a novel chaperone system that rescues previously aggregated proteins. Cell 94, 73–82. 10.1016/S0092-8674(00)81223-4 9674429

[B67] Goate A. Chartier-Harlin M.-C. Mullan M. Brown J. Crawford F. Fidani L.. (1991). Segregation of a missense mutation in the amyloid precursor protein gene with familial Alzheimer's disease. Nature 349, 704–706. 10.1038/349704a0 1671712

[B68] Goedert M. Spillantini M. G. (2006). A century of Alzheimer's disease. Science 314, 777–781. 10.1126/science.1132814 17082447

[B69] Gordon S. Taylor P. R. (2005). Monocyte and macrophage heterogeneity. Nat. Rev. Immunol. 5, 953–964. 10.1038/nri1733 16322748

[B70] Griciuc A. Serrano-Pozo A. Parrado A. R. Lesinski A. N. Asselin C. N. Mullin K.. (2013). Alzheimer's disease risk gene CD33 inhibits microglial uptake of amyloid beta. Neuron 78, 631–643. 10.1016/j.neuron.2013.04.014 23623698 PMC3706457

[B71] Guerreiro R. Wojtas A. Bras J. Carrasquillo M. Rogaeva E. Majounie E.. (2013). TREM2 variants in Alzheimer's disease. N.Engl. J. Med. 368, 117–127. 10.1056/NEJMoa1211851 23150934 PMC3631573

[B72] Hageman J. Rujano M. A. van Waarde M. A. Kakkar V. Dirks R. P. Govorukhina N.. (2010). A DNAJB chaperone subfamily with HDAC-dependent activities suppresses toxic protein aggregation. Mol. Cell 37, 355–369. 10.1016/j.molcel.2010.01.001 20159555

[B73] Hall D. M. Xu L. Drake V. J. Oberley L. W. Oberley T. D. Moseley P. L.. (2000). Aging reduces adaptive capacity and stress protein expression in the liver after heat stress. J. Appl. Physiol. 89, 749–759. 10926662 10.1152/jappl.2000.89.2.749

[B74] Halliday M. Mallucci G. R. (2015). Review: modulating the unfolded protein response to prevent neurodegeneration and enhance memory. Neuropathol. Appl. Neurobiol. 41, 414–427. 10.1111/nan.12211 25556298 PMC5053297

[B75] Halliday M. Radford H. Mallucci G. R. (2014). Prions: generation and spread versus neurotoxicity. J. Biol. Chem. 289, 19862–19868. 10.1074/jbc.R114.568477 24860100 PMC4106307

[B76] Han T. W. Kato M. Xie S. Wu L. C. Mirzaei H. Pei J.. (2012). Cell-free formation of RNA granules: bound RNAs identify features and components of cellular assemblies. Cell 149, 768–779. 10.1016/j.cell.2012.04.016 22579282

[B77] Hanisch U.-K. Kettenmann H. (2007). Microglia: active sensor and versatile effector cells in the normal and pathologic brain. Nat. Neurosci. 10, 1387–1394. 10.1038/nn1997 17965659

[B78] Hartl F. U. Hayer-Hartl M. (2009). Converging concepts of protein folding *in vitro* and *in vivo*. Nat. Struct. Mol. Biol. 16, 574–581. 10.1038/nsmb.1591 19491934

[B79] Hartl F. U. Bracher A. Hayer-Hartl M. (2011). Molecular chaperones in protein folding and proteostasis. Nature 475, 324–332. 10.1038/nature10317 21776078

[B80] Hess N. C. Carlson D. J. Inder J. D. Jesulola E. Mcfarlane J. R. Smart N. A. (2016). Clinically meaningful blood pressure reductions with low intensity isometric handgrip exercise. A randomized trial. Physiol. Res. 65, 461–468. 27070747 10.33549/physiolres.933120

[B81] Hetz C. Mollereau B. (2014). Disturbance of endoplasmic reticulum proteostasis in neurodegenerative diseases. Nat. Rev. Neurosci. 15, 233–249. 10.1038/nrn3689 24619348

[B82] Higuchi S. Arai H. Matsushita S. Matsui T. Kimpara T. Takeda A.. (1998). Mutation in the alpha-synuclein gene and sporadic Parkinson's disease, Alzheimer's disease, and dementia with lewy bodies. Exp. Neurol. 153, 164–166. 10.1006/exnr.1998.6868 9743579

[B83] Hsu A.-L. Murphy C. T. Kenyon C. (2003). Regulation of aging and age-related disease by DAF-16 and heat-shock factor. Science 300, 1142–1145. 10.1126/science.1083701 12750521

[B84] Jankowsky J. L. Slunt H. H. Gonzales V. Savonenko A. V. Wen J. C. Jenkins N. A.. (2005). Persistent amyloidosis following suppression of Abeta production in a transgenic model of Alzheimer disease. PLoS Med. 2:e355. 10.1371/journal.pmed.0020355 16279840 PMC1283364

[B85] Jaunmuktane Z. Mead S. Ellis M. Wadsworth J. D. F. Nicoll A. J. Kenny J.. (2015). Evidence for human transmission of amyloid-β pathology and cerebral amyloid angiopathy. Nature 525, 247–250. 10.1038/nature15369 26354483

[B86] Johnston J. A. Ward C. L. Kopito R. R. (1998). Aggresomes: a cellular response to misfolded proteins. J. Cell Biol. 143, 1883–1898. 10.1083/jcb.143.7.1883 9864362 PMC2175217

[B87] Jonsson T. Stefansson H. Steinberg S. Jonsdottir I. Jonsson P. V. Snaedal J.. (2013). Variant of TREM2 associated with the risk of Alzheimer's disease. N.Engl. J. Med. 368, 107–116. 10.1056/NEJMoa1211103 23150908 PMC3677583

[B88] Jovičić A. Mertens J. Boeynaems S. Bogaert E. Chai N. Yamada S. B.. (2015). Modifiers of C9orf72 dipeptide repeat toxicity connect nucleocytoplasmic transport defects to FTD/ALS. Nat. Neurosci. 18, 1226–1229. 10.1038/nn.4085 26308983 PMC4552077

[B89] Kaganovich D. Kopito R. Frydman J. (2008). Misfolded proteins partition between two distinct quality control compartments. Nature 454, 1088–1095. 10.1038/nature07195 18756251 PMC2746971

[B90] Kakkar V. Månsson C. de Mattos E. P. Bergink S. van der Zwaag M. van Waarde M. A. W. H. (2016). The S/T-rich motif in the DNAJB6 chaperone delays polyglutamine aggregation and the onset of disease in a mouse model. Mol. Cell 62, 272–283. 10.1016/j.molcel.2016.03.017 27151442

[B91] Kakkar V. Meister-Broekema M. Minoia M. Carra S. Kampinga H. H. (2014). Barcoding heat shock proteins to human diseases: looking beyond the heat shock response. Dis. Model. Mech. 7, 421–434. 10.1242/dmm.014563 24719117 PMC3974453

[B92] Kato M. Han T. W. Xie S. Shi K. Du X. Wu L. C.. (2012). Cell-free formation of RNA granules: low complexity sequence domains form dynamic fibers within hydrogels. Cell 149, 753–767. 10.1016/j.cell.2012.04.017 22579281 PMC6347373

[B93] Kaushik S. Cuervo A. M. (2015). Proteostasis and aging. Nat. Med. 21, 1406–1415. 10.1038/nm.4001 26646497

[B94] Keller J. N. Huang F. F. Markesbery W. R. (2000). Decreased levels of proteasome activity and proteasome expression in aging spinal cord. Neuroscience 98, 149–156. 10.1016/S0306-4522(00)00067-1 10858621

[B95] Khan M. R. Li L. Pérez-Sánchez C. Saraf A. Florens L. Slaughter B. D.. (2015). Amyloidogenic oligomerization transforms drosophila Orb2 from a translation repressor to an activator. Cell 163, 1468–1483. 10.1016/j.cell.2015.11.020 26638074 PMC4674814

[B96] Kiffin R. Christian C. Knecht E. Cuervo A. M. (2004). Activation of chaperone-mediated autophagy during oxidative stress. Mol. Biol. Cell 15, 4829–4840. 10.1091/mbc.E04-06-0477 15331765 PMC524731

[B97] Kim M. Lee H. S. LaForet G. McIntyre C. Martin E. J. Chang P.. (1999). Mutant huntingtin expression in clonal striatal cells: dissociation of inclusion formation and neuronal survival by caspase inhibition. J. Neurosci. 19, 964–973. 9920660 10.1523/JNEUROSCI.19-03-00964.1999 PMC6782141

[B98] Kim Y. E. Hipp M. S. Bracher A. Hayer-Hartl M. Ulrich Hartl F. U. (2013). Molecular chaperone functions in protein folding and proteostasis. Annu. Rev. Biochem. 82, 323–355. 10.1146/annurev-biochem-060208-092442 23746257

[B99] Kitada T. Asakawa S. Hattori N. Matsumine H. Yamamura Y. Minoshima S.. (1998). Mutations in the parkin gene cause autosomal recessive juvenile parkinsonism. Nature 392, 605–608. 10.1038/33416 9560156

[B100] Klucken J. Shin Y. Masliah E. Hyman B. T. McLean P. J. (2004). Hsp70 reduces alpha-synuclein aggregation and toxicity. J. Biol. Chem. 279, 25497–25502. 10.1074/jbc.M400255200 15044495

[B101] Knowles T. P. Vendruscolo M. Dobson C. M. (2014). The amyloid state and its association with protein misfolding diseases. Nat. Rev. Mol. Cell Biol. 15, 384–396. 10.1038/nrm3810 24854788

[B102] Koga H. Cuervo A. M. (2011). Chaperone-mediated autophagy dysfunction in the pathogenesis of neurodegeneration. Neurobiol. Dis. 43, 29–37. 10.1016/j.nbd.2010.07.006 20643207 PMC2998583

[B103] Koga H. Kaushik S. Cuervo A. M. (2011). Protein homeostasis and aging: the importance of exquisite quality control. Ageing Res. Rev. 10, 205–215. 10.1016/j.arr.2010.02.001 20152936 PMC2888802

[B104] Kordower J. H. Chu Y. Hauser R. A. Freeman T. B. Olanow C. W. (2008). Lewy body-like pathology in long-term embryonic nigral transplants in Parkinson's disease. Nat. Med. 14, 504–506. 10.1038/nm1747 18391962

[B105] Kwiatkowski T. J. Jr. Bosco D. A. Leclerc A. L. Tamrazian E. Vanderburg C. R. Russ C.. (2009). Mutations in the FUS/TLS gene on chromosome 16 cause familial amyotrophic lateral sclerosis. Science 323, 1205–1208. 10.1126/science.1166066 19251627

[B106] Labbadia J. Morimoto R. I. (2015). The biology of proteostasis in aging and disease. Annu. Rev. Biochem. 84, 435–464. 10.1146/annurev-biochem-060614-033955 25784053 PMC4539002

[B107] Lansbury P. T. Lashuel H. A. (2006). A century-old debate on protein aggregation and neurodegeneration enters the clinic. Nature 443, 774–779. 10.1038/nature05290 17051203

[B108] Lashuel H. A. Lansbury P. T. (2006). Are amyloid diseases caused by protein aggregates that mimic bacterial pore-forming toxins? Q. Rev. Biophys. 39:167. 10.1017/S0033583506004422 16978447

[B109] Lashuel H. A. Hartley D. M. Balakhaneh D. Aggarwal A. Teichberg S. Callaway D. J. E. (2002a). New class of inhibitors of amyloid-beta fibril formation. Implications for the mechanism of pathogenesis in Alzheimer's disease. J. Biol. Chem. 277, 42881–42890. 10.1074/jbc.M206593200 12167652

[B110] Lashuel H. A. Hartley D. Petre B. M. Walz T. Lansbury P. T. (2002b). Neurodegenerative disease: amyloid pores from pathogenic mutations. Nature 418, 291–291. 10.1038/418291a 12124613

[B111] Lashuel H. A. Petre B. M. Wall J. Simon M. Nowak R. J. Walz T.. (2002c). α-synuclein, especially the parkinson's disease-associated mutants, forms pore-like annular and tubular protofibrils. J. Mol. Biol. 322, 1089–1102. 10.1016/S0022-2836(02)00735-0 12367530

[B112] Levy-Lahad E. Wasco W. Poorkaj P. Romano D. M. Oshima J. Pettingell W. H.. (1995). Candidate gene for the chromosome 1 familial Alzheimer's disease locus. Science 269, 973–977. 10.1126/science.7638622 7638622

[B113] Li J.-Y. Englund E. Holton J. L. Soulet D. Hagell P. Lees A. J.. (2008). Lewy bodies in grafted neurons in subjects with Parkinson's disease suggest host-to-graft disease propagation. Nat. Med. 14, 501–503. 10.1038/nm1746 18391963

[B114] Lindberg I. Shorter J. Wiseman R. L. Chiti F. Dickey C. A. McLean P. J. (2015). Chaperones in neurodegeneration. J. Neurosci. 35, 13853–13859. 10.1523/JNEUROSCI.2600-15.2015 26468185 PMC4604223

[B115] Lindquist S. Craig E. A. (1988). The heat-shock proteins. Annu. Rev. Genet. 22, 631–677. 10.1146/annurev.ge.22.120188.003215 2853609

[B116] López-Otín C. Blasco M. A. Partridge L. Serrano M. Kroemer G. (2013). The hallmarks of aging. Cell 153, 1194–1217. 10.1016/j.cell.2013.05.039 23746838 PMC3836174

[B117] Lu J.-X. Qiang W. Yau W.-M. Schwieters C. D. Meredith S. C. Tycko R. (2013). Molecular structure of β-amyloid fibrils in Alzheimer's disease brain tissue. Cell 154, 1257–1268. 10.1016/j.cell.2013.08.035 24034249 PMC3814033

[B118] Luk K. C. Kehm V. M. Zhang B. O'Brien P. Trojanowski J. Q. Lee V. M. Y. (2012b). Intracerebral inoculation of pathological α-synuclein initiates a rapidly progressive neurodegenerative α-synucleinopathy in mice. J. Exp. Med. 209, 975–986. 10.1084/jem.20112457 22508839 PMC3348112

[B119] Luk K. C. Kehm V. Carroll J. Zhang B. Brien P. O. Trojanowski J. Q.. (2012a). Pathological α-synuclein transmission initiates Parkinson-like neurodegeneration in nontransgenic mice. Science 338, 949–954. 10.1126/science.1227157 23161999 PMC3552321

[B120] Madeo F. Zimmermann A. Maiuri M. C. Kroemer G. (2015). Essential role for autophagy in life span extension. J. Clin. Invest. 125, 85–93. 10.1172/JCI73946 25654554 PMC4382258

[B121] Mahul-Mellier A.-L. Vercruysse F. Maco B. Ait-Bouziad N. De Roo M. Muller D.. (2015). Fibril growth and seeding capacity play key roles in α-synuclein-mediated apoptotic cell death. Cell Death Differ. 22, 2107–2122. 10.1038/cdd.2015.79 26138444 PMC4816119

[B122] Maji S. K. Perrin M. H. Sawaya M. R. Jessberger S. Vadodaria K. Rissman R. A.. (2009). Functional amyloids as natural storage of peptide hormones in pituitary secretory granules. Science 325, 328–332. 10.1126/science.1173155 19541956 PMC2865899

[B123] Majumdar A. Cesario W. C. White-Grindley E. Jiang H. Ren F. Khan M. R.. (2012). Critical role of amyloid-like oligomers of Drosophila Orb2 in the persistence of memory. Cell 148, 515–529. 10.1016/j.cell.2012.01.004 22284910

[B124] Månsson C. Arosio P. Hussein R. Kampinga H. H. Hashem R. M. Boelens W. C.. (2014a). Interaction of the molecular chaperone DNAJB6 with growing amyloid-beta 42 (Aβ42) aggregates leads to sub-stoichiometric inhibition of amyloid formation. J. Biol. Chem. 289, 31066–31076. 10.1074/jbc.M114.595124 25217638 PMC4223311

[B125] Månsson C. Kakkar V. Monsellier E. Sourigues Y. Härmark J. Kampinga H. H.. (2014b). DNAJB6 is a peptide-binding chaperone which can suppress amyloid fibrillation of polyglutamine peptides at substoichiometric molar ratios. Cell Stress Chaperones 19, 227–239. 10.1007/s12192-013-0448-5 23904097 PMC3933622

[B126] Marzban L. Trigo-Gonzalez G. Verchere C. B. (2005). Processing of pro-islet amyloid polypeptide in the constitutive and regulated secretory pathways of β Cells. Mol. Endocrinol. 19, 2154–2163. 10.1210/me.2004-0407 15802374

[B127] Masuda-Suzukake M. Nonaka T. Hosokawa M. Oikawa T. Arai T. Akiyama H.. (2013). Prion-like spreading of pathological α-synuclein in brain. Brain 136, 1128–1138. 10.1093/brain/awt037 23466394 PMC3613715

[B128] Maurer-Stroh S. Debulpaep M. Kuemmerer N. Lopez de la Paz M. Martins I. C. Reumers J.. (2010). Exploring the sequence determinants of amyloid structure using position-specific scoring matrices. Nat. Methods 7, 237–242. 10.1038/nmeth.1432 20154676

[B129] McGlinchey R. P. Shewmaker F. McPhie P. Monterroso B. Thurber K. Wickner R. B. (2009). The repeat domain of the melanosome fibril protein Pmel17 forms the amyloid core promoting melanin synthesis. Proc. Natl. Acad. Sci. U.S.A. 106, 13731–13736. 10.1073/pnas.0906509106 19666488 PMC2728962

[B130] Meyer-Luehmann M. Coomaraswamy J. Bolmont T. Kaeser S. Schaefer C. Kilger E.. (2006). Exogenous induction of cerebral beta-amyloidogenesis is governed by agent and host. Science 313, 1781–1784. 10.1126/science.1131864 16990547

[B131] Molliex A. Temirov J. Lee J. Coughlin M. Kanagaraj A. P. Kim H. J.. (2015). Phase separation by low complexity domains promotes stress granule assembly and drives pathological fibrillization. Cell 163, 123–133. 10.1016/j.cell.2015.09.015 26406374 PMC5149108

[B132] Morimoto R. I. (2008). Proteotoxic stress and inducible chaperone networks in neurodegenerative disease and aging. Genes Dev. 22, 1427–1438. 10.1101/gad.1657108 18519635 PMC2732416

[B133] Morley J. F. Morimoto R. I. (2003). Regulation of longevity in *Caenorhabditis elegans* by heat shock factor and molecular chaperones. Mol. Biol. Cell 15, 657–664. 10.1091/mbc.E03-07-0532 14668486 PMC329286

[B134] Mougenot A. L. Nicot S. Bencsik A. Morignat E. Verchère J. Lakhdar L.. (2012). Prion-like acceleration of a synucleinopathy in a transgenic mouse model. Neurobiol. Aging 33, 2225–2228. 10.1016/j.neurobiolaging.2011.06.022 21813214

[B135] Murrell J. Farlow M. Ghetti B. Benson M. D. (1991). A mutation in the amyloid precursor protein associated with hereditary Alzheimer's disease. Science 254, 97–99. 10.1126/science.1925564 1925564

[B136] Nillegoda N. B. Bukau B. (2015). Metazoan Hsp70-based protein disaggregases: emergence and mechanisms. Front. Mol. Biosci. 2:57. 10.3389/fmolb.2015.00057 26501065 PMC4598581

[B137] Oesch B. Westaway D. Wälchli M. McKinley M. P. Kent S. B. Aebersold R.. (1985). A cellular gene encodes scrapie PrP 27-30 protein. Cell 40, 735–746. 10.1016/0092-8674(85)90333-2 2859120

[B138] Ohtake H. Limprasert P. Fan Y. Onodera O. Kakita A. Takahashi H.. (2004). Beta-synuclein gene alterations in dementia with Lewy bodies. Neurology 63, 805–811. 10.1212/01.WNL.0000139870.14385.3C 15365127 PMC1808539

[B139] Olzscha H. Schermann S. M. Woerner A. C. Pinkert S. Hecht M. H. Tartaglia G. G.. (2011). Amyloid-like aggregates sequester numerous metastable proteins with essential cellular functions. Cell 144, 67–78. 10.1016/j.cell.2010.11.050 21215370

[B140] Opie E. L. (1901). The relation of diabetes mellitus to lesions of the pancreas. Hyaline degeneration of the islands of langerhans. J. Exp. Med. 5, 527–540. 10.1084/jem.5.5.527 19866956 PMC2118021

[B141] Oropesa-Nuñez R. Seghezza S. Dante S. Diaspro A. Cascella R. Cecchi C.. (2016). Interaction of toxic and non-toxic HypF-N oligomers with lipid bilayers investigated at high resolution with atomic force microscopy. Oncotarget 7, 20–23. 10.18632/oncotarget.10449 27391440 PMC5216700

[B142] Pahlavani M. A. Harris M. D. Moore S. A. Weindruch R. Richardson A. (1995). The expression of heat shock protein 70 decreases with age in lymphocytes from rats and rhesus monkeys. Exp. Cell Res. 218, 310–318. 10.1006/excr.1995.1160 7737368

[B143] Park S.-H. Kukushkin Y. Gupta R. Chen T. Konagai A. Hipp M. S.. (2013). PolyQ proteins interfere with nuclear degradation of cytosolic proteins by sequestering the Sis1p chaperone. Cell 154, 134–145. 10.1016/j.cell.2013.06.003 23791384

[B144] Patel A. Lee H. O. Jawerth L. Maharana S. Drechsel D. Alberti S.. (2015). A liquid-to-solid phase transition of the ALS protein FUS accelerated by disease mutation. Cell 162, 1066–1077. 10.1016/j.cell.2015.07.047 26317470

[B145] Paz Gavilán M. Vela J. Castaño A. Ramos B. del Río J. C. Vitorica J.. (2006). Cellular environment facilitates protein accumulation in aged rat hippocampus. Neurobiol. Aging 27, 973–982. 10.1016/j.neurobiolaging.2005.05.010 15964666

[B146] Perng M. D. Muchowski P. J. van Den IJssel P. Wu G. J. Hutchesont A. M. Clark J. I.. (1999). The cardiomyopathy and lens cataract mutation in alphaB-crystallin alters its protein structure, chaperone activity, and interaction with intermediate filaments *in vitro*. J. Biol. Chem. 274, 33235–33243. 10.1074/jbc.274.47.33235 10559197

[B147] Perry V. H. Nicoll J. A. Holmes C. (2010). Microglia in neurodegenerative disease. Nat. Rev. Neurol. 6, 193–201. 10.1038/nrneurol.2010.17 20234358

[B148] Pierce A. Podlutskaya N. Halloran J. J. Hussong S. A. Lin P.-Y. Burbank R.. (2013). Over-expression of heat shock factor 1 phenocopies the effect of chronic inhibition of TOR by rapamycin and is sufficient to ameliorate Alzheimer's-like deficits in mice modeling the disease. J. Neurochem. 124, 880–893. 10.1111/jnc.12080 23121022 PMC6762020

[B149] Pierce A. Wei R. Halade D. Yoo S.-E. Ran Q. Richardson A. (2010). A Novel mouse model of enhanced proteostasis: full-length human heat shock factor 1 transgenic mice. Biochem. Biophys. Res. Commun. 402, 59–65. 10.1016/j.bbrc.2010.09.111 20920476

[B150] Polymeropoulos M. H. Lavedan C. Leroy E. Ide S. E. Dehejia A. Dutra A.. (1997). Mutation in the α-synuclein gene identified in families with Parkinson's disease. Science 276, 2045–2047. 10.1126/science.276.5321.2045 9197268

[B151] Radwan M. Wood R. J. Sui X. Hatters D. M. (2017). When proteostasis goes bad: protein aggregation in the cell. IUBMB Life. 69, 49–54. 10.1002/iub.1597 28066979

[B152] Ramaswami M. Taylor J. P. Parker R. (2013). Altered ribostasis: RNA-protein granules in degenerative disorders. Cell 154, 727–736. 10.1016/j.cell.2013.07.038 23953108 PMC3811119

[B153] Rampelt H. Kirstein-Miles J. Nillegoda N. B. Chi K. Scholz S. R. Morimoto R. I.. (2012). Metazoan Hsp70 machines use Hsp110 to power protein disaggregation. EMBO J. 31, 4221–4235. 10.1038/emboj.2012.264 22990239 PMC3492728

[B154] Renton A. E. Majounie E. Waite A. Simón-Sánchez J. Rollinson S. Gibbs J. R.. (2011). A hexanucleotide repeat expansion in C9ORF72 is the cause of chromosome 9p21-linked ALS-FTD. Neuron 72, 257–268. 10.1016/j.neuron.2011.09.010 21944779 PMC3200438

[B155] Rhein V. Song X. Wiesner A. Ittner L. M. Baysang G. Meier F.. (2009). Amyloid-beta and tau synergistically impair the oxidative phosphorylation system in triple transgenic Alzheimer's disease mice. Proc. Natl. Acad. Sci. U.S.A. 106, 20057–20062. 10.1073/pnas.0905529106 19897719 PMC2774257

[B156] Ridley R. M. Baker H. F. Windle C. P. Cummings R. M. (2006). Very long term studies of the seeding of beta-amyloidosis in primates. J. Neural. Transm. 113, 1243–1251. 10.1007/s00702-005-0385-2 16362635

[B157] Rogaev E. I. (1995). Familial Alzheimer's disease in kindreds with missense mutations in a gene on chromosome 1 related to the Alzheimer's disease type 3 gene. Nature 376, 775–778. 10.1038/376775a0 7651536

[B158] Rosen D. R. Siddique T. Patterson D. Figlewicz D. A. Sapp P. Hentati A.. (1993). Mutations in Cu/Zn superoxide dismutase gene are associated with familial amyotrophic lateral sclerosis. Nature 362, 59–62. 10.1038/362059a0 8446170

[B159] Rothenberg C. Srinivasan D. Mah L. Kaushik S. Peterhoff C. M. Ugolino J.. (2010). Ubiquilin functions in autophagy and is degraded by chaperone-mediated autophagy. Hum. Mol. Genet. 19, 3219–3232. 10.1093/hmg/ddq231 20529957 PMC2908472

[B160] Sanders D. W. Kaufman S. K. DeVos S. L. Sharma A. M. Mirbaha H. Li A.. (2014). Distinct tau prion strains propagate in cells and mice and define different tauopathies. Neuron 82, 1271–1288. 10.1016/j.neuron.2014.04.047 24857020 PMC4171396

[B161] Scheper W. Hoozemans J. J. (2015). The unfolded protein response in neurodegenerative diseases: a neuropathological perspective. Acta Neuropathol. 130, 315–331. 10.1007/s00401-015-1462-8 26210990 PMC4541706

[B162] Scherzinger E. Sittler A. Schweiger K. Heiser V. Lurz R. Hasenbank R.. (1999). Self-assembly of polyglutamine-containing huntingtin fragments into amyloid-like fibrils: implications for Huntington's disease pathology. Proc. Natl. Acad. Sci. U.S.A. 96, 4604–4609. 10.1073/pnas.96.8.4604 10200309 PMC16379

[B163] Sharma S. K. Priya S. (2016). Expanding role of molecular chaperones in regulating α-synuclein misfolding; implications in Parkinson's disease. Cell. Mol. Life Sci. 74, 617–629. 10.1007/s00018-016-2340-9 27522545 PMC11107554

[B164] Shelkovnikova T. A. Robinson H. K. Southcombe J. A. Ninkina N. Buchman V. L. (2014). Multistep process of FUS aggregation in the cell cytoplasm involves RNA-dependent and RNA-independent mechanisms. Hum. Mol. Genet. 23, 5211–5226. 10.1093/hmg/ddu243 24842888 PMC4159159

[B165] Sherrington R. Rogaev E. I. Liang Y. Rogaeva E. A. Levesque G. Ikeda M.. (1995). Cloning of a gene bearing missense mutations in early-onset familial Alzheimer's disease. Nature 375, 754–760. 10.1038/375754a0 7596406

[B166] Shin J. Y. Fang Z. H. Yu Z. X. Wang C. E. Li S. H. Li X. J. (2005). Expression of mutant huntingtin in glial cells contributes to neuronal excitotoxicity. J. Cell Biol. 171, 1001–1012. 10.1083/jcb.200508072 16365166 PMC2171327

[B167] Shorter J. (2011). The mammalian disaggregase machinery: Hsp110 synergizes with Hsp70 and Hsp40 to catalyze protein disaggregation and reactivation in a cell-free system. PLoS ONE 6:e26319. 10.1371/journal.pone.0026319 22022600 PMC3194798

[B168] Singh R. Kølvraa S. Bross P. Jensen U. B. Gregersen N. Tan Q.. (2006). Reduced heat shock response in human mononuclear cells during aging and its association with polymorphisms in HSP70 genes. Cell Stress Chaperones 11, 208–215. 10.1379/CSC-184R.1 17009593 PMC1576475

[B169] Singleton A. B. Farrer M. Johnson J. Singleton A. Hague S. Kachergus J.. (2003). alpha-Synuclein locus triplication causes Parkinson's disease. Science 302:841. 10.1126/science.1090278 14593171

[B170] Sipe J. D. Cohen A. S. (2000). Review: history of the amyloid fibril. J. Struct. Biol. 130, 88–98. 10.1006/jsbi.2000.4221 10940217

[B171] Sokolowski F. Modler A. J. Masuch R. Zirwer D. Baier M. Lutsch G.. (2003). Formation of critical oligomers is a key event during conformational transition of recombinant syrian hamster prion protein. J. Biol. Chem. 278, 40481–40492. 10.1074/jbc.M304391200 12917432

[B172] Sontag E. M. Vonk W. I. Frydman J. (2014). Sorting out the trash: the spatial nature of eukaryotic protein quality control. Curr. Opin. Cell Biol. 26, 139–146. 10.1016/j.ceb.2013.12.006 24463332 PMC4204729

[B173] Specht S. Miller S. B. Mogk A. Bukau B. (2011). Hsp42 is required for sequestration of protein aggregates into deposition sites in *Saccharomyces cerevisiae*. J. Cell Biol. 195, 617–629. 10.1083/jcb.201106037 22065637 PMC3257523

[B174] Stefani M. Dobson C. M. (2003). Protein aggregation and aggregate toxicity: new insights into protein folding, misfolding diseases and biological evolution. J. Mol. Med. 81, 678–699. 10.1007/s00109-003-0464-5 12942175

[B175] Stöckl M. T. Zijlstra N. Subramaniam V. (2013). α-Synuclein oligomers: an amyloid pore? Insights into mechanisms of α-synuclein oligomer-lipid interactions. Mol. Neurobiol. 47, 613–621. 10.1007/s12035-012-8331-4 22956232

[B176] Stöhr J. Watts J. C. Mensinger Z. L. Oehler A. Grillo S. K. DeArmond S. J.. (2012). Purified and synthetic Alzheimer's amyloid beta (Aβ) prions. Proc. Natl. Acad. Sci. U.S.A. 109, 11025–11030. 10.1073/pnas.1206555109 22711819 PMC3390876

[B177] Taylor R. C. (2016). Aging and the UPR(ER). Brain Res. 1648, 588–593. 10.1016/j.brainres.2016.04.017 27067187

[B178] Taylor R. C. Dillin A. (2013). XBP-1 is a cell-nonautonomous regulator of stress resistance and longevity. Cell 153, 1435–1447. 10.1016/j.cell.2013.05.042 23791175 PMC4771415

[B179] Torrente M. P. Shorter J. (2013). The metazoan protein disaggregase and amyloid depolymerase system: Hsp110, Hsp70, Hsp40, and small heat shock proteins. Prion 7, 457–463. 10.4161/pri.27531 24401655 PMC4201613

[B180] Tyedmers J. Mogk A. Bukau B. (2010). Cellular strategies for controlling protein aggregation. Nat. Rev. Mol. Cell Biol. 11, 777–788. 10.1038/nrm2993 20944667

[B181] Uversky V. N. (2017). Intrinsically disordered proteins in overcrowded milieu: membrane-less organelles, phase separation, and intrinsic disorder. Curr. Opin. Struct. Biol. 44, 18–30. 10.1016/j.sbi.2016.10.015 27838525

[B182] Valente E. M. Bentivoglio A. R. Dixon P. H. Ferraris A. Ialongo T. Frontali M.. (2001). Localization of a novel locus for autosomal recessive early-onset parkinsonism, PARK6, on human chromosome 1p35-p36. Am. J. Hum. Genet. 68, 895–900. 10.1086/319522 11254447 PMC1275643

[B183] van Ham T. J. Holmberg M. A. van der Goot A. T. Teuling E. Garcia-Arencibia M. Kim H. E.. (2010). Identification of MOAG-4/SERF as a regulator of age-related proteotoxicity. Cell 142, 601–612. 10.1016/j.cell.2010.07.020 20723760

[B184] Ventura S. Zurdo J. Narayanan S. Parreño M. Mangues R. Reif B.. (2004). Short amino acid stretches can mediate amyloid formation in globular proteins: the Src homology 3 (SH3) case. Proc. Natl. Acad. Sci. U.S.A. 101, 7258–7263. 10.1073/pnas.0308249101 15123800 PMC409906

[B185] Vicart P. Caron A. Guicheney P. Li Z. Prévost M. C. Faure A.. (1998). A missense mutation in the alphaB-crystallin chaperone gene causes a desmin-related myopathy. Nat. Genet. 20, 92–95. 10.1038/1765 9731540

[B186] Wacker J. L. Zareie M. H. Fong H. Sarikaya M. Muchowski P. J. (2004). Hsp70 and Hsp40 attenuate formation of spherical and annular polyglutamine oligomers by partitioning monomer. Nat. Struct. Mol. Biol. 11, 1215–1222. 10.1038/nsmb860 15543156

[B187] Walker G. A. White T. M. McColl G. Jenkins N. L. Babich S. Candido E. P.. (2001). Heat shock protein accumulation is upregulated in a long-lived mutant of Caenorhabditis elegans. J. Gerontol. A Biol. Sci. Med. Sci. 56, 281–287. 10.1093/gerona/56.7.B281 11445592

[B188] Watt B. van Niel G. Raposo G. Marks M. S. (2013). PMEL: a pigment cell-specific model for functional amyloid formation. Pigment Cell Melanoma Res. 26, 300–315. 10.1111/pcmr.12067 23350640 PMC3633693

[B189] Watts J. C. Condello C. Stöhr J. Oehler A. Lee J. DeArmond S. J.. (2014). Serial propagation of distinct strains of Aβ prions from Alzheimer's disease patients. Proc. Natl. Acad. Sci. U.S.A. 111, 10323–10328. 10.1073/pnas.1408900111 24982139 PMC4104857

[B190] Westermark G. T. Westermark P. (2013). Islet amyloid polypeptide and diabetes. Curr. Protein Pept. Sci. 14, 330–337. 10.2174/13892037113149990050 23745697

[B191] Westermark P. Engström U. Westermark G. T. Johnson K. H. Permerth J. Betsholtz C. (1989). Islet amyloid polypeptide (IAPP) and pro-IAPP immunoreactivity in human islets of Langerhans. Diabetes Res. Clin. Pract. 7, 219–226. 10.1016/0168-8227(89)90008-9 2691219

[B192] Wilhelmsen K. C. Lynch T. Pavlou E. Higgins M. Nygaard T. G. (1994). Localization of disinhibition-dementia-parkinsonism-amyotrophy complex to 17q21-22. Am. J. Hum. Genet. 55, 1159–1165. 7977375 PMC1918447

[B193] Winkler J. Tyedmers J. Bukau B. Mogk A. (2012). Chaperone networks in protein disaggregation and prion propagation. J. Struct. Biol. 179, 152–160. 10.1016/j.jsb.2012.05.002 22580344

[B194] Wiseman F. K. Al-Janabi T. Hardy J. Karmiloff-Smith A. Nizetic D. Tybulewicz V. L.. (2015). A genetic cause of Alzheimer disease: mechanistic insights from Down syndrome. Nat. Rev. Neurosci. 16, 564–574. 10.1038/nrn3983 26243569 PMC4678594

[B195] Woerner A. C. Frottin F. Hornburg D. Feng L. R. Meissner F. Patra M.. (2016). Cytoplasmic protein aggregates interfere with nucleocytoplasmic transport of protein and RNA. Science 351, 173–176. 10.1126/science.aad2033 26634439

[B196] Ye L. Fritschi S. K. Schelle J. Obermüller U. Degenhardt K. Kaeser S. A.. (2015). Persistence of Aβ seeds in APP null mouse brain. Nat. Neurosci. 18, 16–19. 10.1038/nn.4117 26352792

[B197] Yerbury J. J. Ooi L. Dillin A. Saunders D. N. Hatters D. M. Beart P. M.. (2016). Walking the tightrope: proteostasis and neurodegenerative disease. J. Neurochem. 137, 489–505. 10.1111/jnc.13575 26872075

[B198] Zhang K. Donnelly C. J. Haeusler A. R. Grima J. C. Machamer J. B. Steinwald P.. (2015). The C9orf72 repeat expansion disrupts nucleocytoplasmic transport. Nature 525, 56–61. 10.1038/nature14973 26308891 PMC4800742

[B199] Zimprich A. Biskup S. Leitner P. Lichtner P. Farrer M. Lincoln S.. (2004). Mutations in LRRK2 cause autosomal-dominant parkinsonism with pleomorphic pathology. Neuron 44, 601–607. 10.1016/j.neuron.2004.11.005 15541309

